# Supplementary material for: Effectiveness of Group Problem Management Plus, a brief psychological intervention for adults affected by humanitarian disasters in Nepal: A cluster randomized controlled trial
Source: PLoS Med. 2021 Jun 17;18(6):e1003621. doi: 10.1371/journal.pmed.1003621 (PMC8211182; doi:10.1371/journal.pmed.1003621)
Supplement: S1 Text — RTC, Reducing Tension Checklist. (DOCX) [file pmed.1003621.s001.docx]

**Reducing Tension Checklist (RTC)**

People do different things to solve their problems and reduce the stress and tension in their lives and the lives of friends and family. Please let us know if you do any of the following activities.

1. In the past month, how much have you **helped** friends or family with difficulties/problems such as child care, house work, office or school work, finances, transportation, etc.?

0=Not at all

  1=A little/rarely (once or twice in the past month)^[[1]](#footnote-1)^

               2=Sometimes (about once a week)

               3=Most of the time (a few times per week)

               4=All the time (almost every day)

1. In the past month, how much have you **asked your** friends or family, to help with difficulties/problems such as child care, house work, office or school work, finances, transportation, etc.?

0=Not at all

  1=A little/rarely (once or twice in the past month)

               2=Sometimes (about once a week)

               3=Most of the time (a few times per week)

               4=All the time (almost every day)

1. In the past month, how much have **you listened** **to your friends or family** when they talked about stress they are experiencing?

0=Not at all

  1=A little/rarely (once or twice in the past month)

               2=Sometimes (about once a week)

               3=Most of the time (a few times per week)

               4=All the time (almost every day)

1. In the past month, how much have **you told friends or family** about stress in your life?

0=Not at all

  1=A little/rarely (once or twice in the past month)

               2=Sometimes (about once a week)

               3=Most of the time (a few times per week)

               4=All the time (almost every day)

1. In the past month, how much time did you **spend meeting with and talking to your relatives and friends**?

0=Not at all

  1=A little/rarely (once or twice in the past month)

               2=Sometimes (about once a week)

               3=Most of the time (a few times per week)

               4=All the time (almost every day)

1. In the past month, how much have you tried to **do deep or slow breathing** to manage your stress?

0=Not at all

               1=A little/rarely (once or twice in the past month)

               2=Sometimes (about once a week)

               3=Most of the time (a few times per week)

               4=All the time (almost every day)

1. In the past month, when you are feeling down or having low energy, how much have **you done something that energizes you help get started working again** (including household work, professional work, school work, etc.)?

0=Not at all

  1=A little/rarely (once or twice in the past month)

               2=Sometimes (about once a week)

               3=Most of the time (a few times per week)

               4=All the time (almost every day)

1. In the past month, how much have you tried to **distinguish between which of your problems can be solved and which of your problems cannot be solved**?

0=Not at all

  1=A little/rarely (once or twice in the past month)

               2=Sometimes (about once a week)

               3=Most of the time (a few times per week)

               4=All the time (almost every day)

1. In the past month, how often have you tried to **brainstorm different ways to manage your problems**?

0=Not at all

  1=A little/rarely (once or twice in the past month)

               2=Sometimes (about once a week)

               3=Most of the time (a few times per week)

               4=All the time (almost every day)

1. In the past month, how much have you **separated/broken down your problems into small manageable steps** rather than trying to solve everything at once?

0=Not at all

  1=A little/rarely (once or twice in the past month)

               2=Sometimes (about once a week)

               3=Most of the time (a few times per week)

               4=All the time (almost every day)

1. The text in parentheses with time anchors are optional and were not used in the Group PM+ trial in Nepal. [↑](#footnote-ref-1)
