# Supplementary material for: Effectiveness of Group Problem Management Plus, a brief psychological intervention for adults affected by humanitarian disasters in Nepal: A cluster randomized controlled trial
Source: PLoS Med. 2021 Jun 17;18(6):e1003621. doi: 10.1371/journal.pmed.1003621 (PMC8211182; doi:10.1371/journal.pmed.1003621)
Supplement: S2 Text — CONSORT, Consolidated Standards of Reporting Trials. (DOCX) [file pmed.1003621.s002.docx]

**CONSORT Table 1: CONSORT 2010 checklist of information to include when reporting a cluster randomised trial**

| Section/Topic | Item No | Standard Checklist item | Extension for cluster designs | Location |
| --- | --- | --- | --- | --- |
| Title and abstract | | | |  |
|  | 1a | Identification as a randomised trial in the title | Identification as a cluster randomised trial in the title | Title |
|  | 1b | Structured summary of trial design, methods, results, and conclusions (for specific guidance see CONSORT for abstracts)^[[1]](#endnote-1),^^[[2]](#endnote-2)^ | See CONSORT Table 2 below. | See CONSORT Table 2 below. |
| Introduction | | | |  |
| Background and objectives | 2a | Scientific background and explanation of rationale | Rationale for using a cluster design | Protocol paper (p. 3) |
|  | 2b | Specific objectives or hypotheses | Whether objectives pertain to the cluster level, the individual participant level or both | Final paragraph of Introduction |
| Methods | | | |  |
| Trial design | 3a | Description of trial design (such as parallel, factorial) including allocation ratio | Definition of cluster and description of how the design features apply to the clusters | First paragraph of ‘Randomization and masking’ & protocol paper |
|  | 3b | Important changes to methods after trial commencement (such as eligibility criteria), with reasons |  | ‘Participants’ section |
| Participants | 4a | Eligibility criteria for participants | Eligibility criteria for clusters | ‘Participants’ section |
|  | 4b | Settings and locations where the data were collected |  | ‘Procedures’ section |
| Interventions | 5 | The interventions for each group with sufficient details to allow replication, including how and when they were actually administered | Whether interventions pertain to the cluster level, the individual participant level or both | ‘Intervention’ section |
| Outcomes | 6a | Completely defined pre-specified primary and secondary outcome measures, including how and when they were assessed | Whether outcome measures pertain to the cluster level, the individual participant level or both | ‘Instruments’ section & protocol paper |
|  | 6b | Any changes to trial outcomes after the trial commenced, with reasons |  | ‘Instruments’ section |
| Sample size | 7a | How sample size was determined | Method of calculation, number of clusters(s) (and whether equal or unequal cluster sizes are assumed), cluster size, a coefficient of intracluster correlation (ICC or *k*), and an indication of its uncertainty | ‘Analysis’ section |
|  | 7b | When applicable, explanation of any interim analyses and stopping guidelines |  | ‘Analysis’ section |
| Randomisation: | | | |  |
| Sequence generation | 8a | Method used to generate the random allocation sequence |  | ‘Randomization and masking’ section |
|  | 8b | Type of randomisation; details of any restriction (such as blocking and block size) | Details of stratification or matching if used | ‘Randomization and masking’ section |
| Allocation concealment mechanism | 9 | Mechanism used to implement the random allocation sequence (such as sequentially numbered containers), describing any steps taken to conceal the sequence until interventions were assigned | Specification that allocation was based on clusters rather than individuals and whether allocation concealment (if any) was at the cluster level, the individual participant level or both | ‘Randomization and masking’ section |
| Implementation | 10 | Who generated the random allocation sequence, who enrolled participants, and who assigned participants to interventions | Replace by 10a, 10b and 10c |  |
|  | 10a |  | Who generated the random allocation sequence, who enrolled clusters, and who assigned clusters to interventions | ‘Randomization and masking’ section and protocol paper |
|  | 10b |  | Mechanism by which individual participants were included in clusters for the purposes of the trial (such as complete enumeration, random sampling) | ‘Randomization and masking’ and ‘Procedures’ sections and protocol paper |
|  | 10c |  | From whom consent was sought (representatives of the cluster, or individual cluster members, or both), and whether consent was sought before or after randomisation | ‘Procedures’ section and protocol paper |
|  |  |  |  |  |
| Blinding | 11a | If done, who was blinded after assignment to interventions (for example, participants, care providers, those assessing outcomes) and how |  | ‘Randomization and masking’ section |
|  | 11b | If relevant, description of the similarity of interventions |  | ‘Interventions’ section |
| Statistical methods | 12a | Statistical methods used to compare groups for primary and secondary outcomes | How clustering was taken into account | ‘Analysis’ section |
|  | 12b | Methods for additional analyses, such as subgroup analyses and adjusted analyses |  | ‘Analysis’ section |
| Results | | | |  |
| Participant flow (a diagram is strongly recommended) | 13a | For each group, the numbers of participants who were randomly assigned, received intended treatment, and were analysed for the primary outcome | For each group, the numbers of clusters that were randomly assigned, received intended treatment, and were analysed for the primary outcome | Figure 1 & supplemental tables |
|  | 13b | For each group, losses and exclusions after randomisation, together with reasons | For each group, losses and exclusions for both clusters and individual cluster members | Figure 1 & supplemental tables |
| Recruitment | 14a | Dates defining the periods of recruitment and follow-up |  | ‘Participant flow and recruitment’ section |
|  | 14b | Why the trial ended or was stopped |  | ‘Procedures’ section |
| Baseline data | 15 | A table showing baseline demographic and clinical characteristics for each group | Baseline characteristics for the individual and cluster levels as applicable for each group | Table 1 & supplemental tables |
| Numbers analysed | 16 | For each group, number of participants (denominator) included in each analysis and whether the analysis was by original assigned groups | For each group, number of clusters included in each analysis | Figure 1 & supplemental tables |
| Outcomes and estimation | 17a | For each primary and secondary outcome, results for each group, and the estimated effect size and its precision (such as 95% confidence interval) | Results at the individual or cluster level as applicable and a coefficient of intracluster correlation (ICC or k) for each primary outcome | Tables 3-5 & supplemental tables |
|  | 17b | For binary outcomes, presentation of both absolute and relative effect sizes is recommended |  | Tables 3-5 & supplemental tables |
| Ancillary analyses | 18 | Results of any other analyses performed, including subgroup analyses and adjusted analyses, distinguishing pre-specified from exploratory |  | Tables 3-5 & supplemental tables |
| Harms | 19 | All important harms or unintended effects in each group (for specific guidance see CONSORT for harms^[[3]](#endnote-3)^) |  | ‘Harms’ section |
| Discussion | | | |  |
| Limitations | 20 | Trial limitations, addressing sources of potential bias, imprecision, and, if relevant, multiplicity of analyses |  | Final paragraph of Discussion |
| Generalisability | 21 | Generalisability (external validity, applicability) of the trial findings | Generalisability to clusters and/or individual participants (as relevant) | Discussion section |
| Interpretation | 22 | Interpretation consistent with results, balancing benefits and harms, and considering other relevant evidence |  | Discussion section |
| Other information | | |  |  |
| Registration | 23 | Registration number and name of trial registry |  | **ClinicalTrials.gov NCT03747055** |
| Protocol | 24 | Where the full trial protocol can be accessed, if available |  | https://trialsjournal.biomedcentral.com/ articles/10.1186/s13063-020-04263-9 |
| Funding | 25 | Sources of funding and other support (such as supply of drugs), role of funders |  | USAID OFDA funded this study in a grant to the World Health Organization. BAK is supported by the US National Institute of Mental Health (K01MH104310, R01MH120649). The funders played no role in the design and conduct of the study; collection, management, analysis, and interpretation of the data; preparation, review, or approval of the manuscript; and decision to submit the manuscript for publication. The authors alone are responsible for the views expressed in this publication and they do not necessarily represent the decisions, policy or views of the World Health Organization. |

** Note: page numbers optional depending on journal requirements*

**CONSORT Table 2: Extension of CONSORT for abstracts to reports of cluster randomised trials**

| Item | Standard Checklist item | Extension for cluster trials | Location in abstract |
| --- | --- | --- | --- |
| Title | Identification of study as randomised | Identification of study as cluster randomised | Title |
| Trial design | Description of the trial design (e.g. parallel, cluster, non-inferiority) |  | Methods and findings |
| Methods |  |  |  |
| Participants | Eligibility criteria for participants and the settings where the data were collected | Eligibility criteria for clusters | Methods and findings |
| Interventions | Interventions intended for each group |  | Methods and findings |
| Objective | Specific objective or hypothesis | Whether objective or hypothesis pertains to the cluster level, the individual participant level or both | Methods and findings |
| Outcome | Clearly defined primary outcome for this report | Whether the primary outcome pertains to the cluster level, the individual participant level or both | Methods and findings |
| Randomization | How participants were allocated to interventions | How clusters were allocated to interventions | Methods and findings |
| Blinding (masking) | Whether or not participants, care givers, and those assessing the outcomes were blinded to group assignment |  | Methods and findings |
| Results |  |  |  |
| Numbers randomized | Number of participants randomized to each group | Number of clusters randomized to each group | Methods and findings |
| Numbers analysed | Number of participants analysed in each group | Number of clusters analysed in each group | Methods and findings |
| Outcome | For the primary outcome, a result for each group and the estimated effect size and its precision | Results at the cluster or individual participant level as applicable for each primary outcome | Methods and findings |
| Harms | Important adverse events or side effects |  | Methods and findings |
| Conclusions | General interpretation of the results |  | ‘Conclusions’ section |
| Trial registration | Registration number and name of trial register |  | ClinicalTrials.gov NCT03747055 |
| Funding | Source of funding |  | ‘Funding’ section of publication |
|  |  |  |  |

**REFERENCES**

1. Hopewell S, Clarke M, Moher D, Wager E, Middleton P, Altman DG, et al. CONSORT for reporting randomised trials in journal and conference abstracts. *Lancet* 2008, 371:281-283 [↑](#endnote-ref-1)
2. Hopewell S, Clarke M, Moher D, Wager E, Middleton P, Altman DG at al (2008) CONSORT for reporting randomized controlled trials in journal and conference abstracts: explanation and elaboration. *PLoS Med* 5(1): e20 [↑](#endnote-ref-2)
3. Ioannidis JP, Evans SJ, Gotzsche PC, O'Neill RT, Altman DG, Schulz K, Moher D. Better reporting of harms in randomized trials: an extension of the CONSORT statement. *Ann Intern Med* 2004; 141(10):781-788. [↑](#endnote-ref-3)
