# Supplementary material for: Effectiveness of Group Problem Management Plus, a brief psychological intervention for adults affected by humanitarian disasters in Nepal: A cluster randomized controlled trial
Source: PLoS Med. 2021 Jun 17;18(6):e1003621. doi: 10.1371/journal.pmed.1003621 (PMC8211182; doi:10.1371/journal.pmed.1003621)
Supplement: S3 Text — (DOCX) [file pmed.1003621.s003.docx]

**Supplemental Analysis Tables**

**Table A. Reasons for ineligibility and dropout** (Supplemental Table to Figure 1. CONSORT Participant Flowchart)

| \| **Reasons for non-eligibility based on screening by arm** \| Control \| PM+ \| Total \| \| --- \| --- \| --- \| --- \| \| (N = 845) \| (N = 566) \| (N = 1411) \| |
| --- | --- | --- | --- | --- | --- | --- | --- |
| \|  \|  \|  \|  \| \| --- \| --- \| --- \| --- \| \| Absence of Heart-Mind problems \| 45 (5.3%) \| 31 (5.5%) \| 76 (5.4%) \| \| WHO Disability Adjustment Schedule (WHODAS<16) \| 398 (47.1%) \| 259 (45.8%) \| 657 (46.6%) \| \| Alcohol Use Disorder Identifiation Test (AUDIT>16) \| 26 (3.1%) \| 28 (4.9%) \| 54 (3.8%) \| \| Absence of Heart-Mind problems & WHO Disability Adjustment Schedule (WHODAS<16) \| 311 (36.8%) \| 200 (35.3%) \| 511 (36.2%) \| \| Absence of Heart-Mind problems & Alcohol Use Disorder Identification Test (AUDIT>16) \| 2 (0.2%) \| 1 (0.2%) \| 3 (0.2%) \| \| WHO Disability Adjustment Schedule (WHODAS<16) & Alcohol Use Disorder Identification Test (AUDIT>16) \| 33 (3.9%) \| 27 (4.8%) \| 60 (4.3%) \| \| Absence of Heart-Mind problems & WHO Disability Adjustment Schedule (WHODAS<16) & Alcohol Use Disorder Identification Test (AUDIT>16) \| 30 (3.6%) \| 20 (3.5%) \| 50 (3.5%) \| |
| \| **Reasons for droup at baseline by arm** \| Control \| PM+ \| Total \| \| --- \| --- \| --- \| --- \| \|  \| (N = 18) \| (N = 14) \| (N = 32) \| |
| \|  \|  \|  \|  \| \| --- \| --- \| --- \| --- \| \| Death \| 2 (11.1%) \| 0 (0.0%) \| 2 (6.2%) \| \| Communication issue \| 1 (5.6%) \| 2 (14.3%) \| 3 (9.4%) \| \| Refusal for family meeting \| 5 (27.8%) \| 3 (21.4%) \| 8 (25.0%) \| \| Refusal for Baseline \| 4 (22.2%) \| 3 (21.4%) \| 7 (21.9%) \| \| Unavailable \| 5 (27.8%) \| 4 (28.6%) \| 9 (28.1%) \| \| Data lost \| 0 (0.0%) \| 1 (7.1%) \| 1 (3.1%) \| \| Severe mental health issue \| 0 (0.0%) \| 1 (7.1%) \| 1 (3.1%) \| \| Physical sickness \| 1 (5.6%) \| 0 (0.0%) \| 1 (3.1%) \| |
| \| **Reasons for dropout/missing at midline by arm** \| Control \| PM+ \| Total \| \| --- \| --- \| --- \| --- \| \| (N = 5) \| (N = 2) \| (N = 7) \| |
| \|  \|  \|  \|  \| \| --- \| --- \| --- \| --- \| \| Refusal for Midline \| 1 (20.0%) \| 1 (50.0%) \| 2 (28.6%) \| \| Unavailable \| 2 (40.0%) \| 1 (50.0%) \| 3 (42.9%) \| \| Severe mental health issue \| 2 (40.0%) \| 0 (0.0%) \| 2 (28.6%) \| |
| \| **Reasons for dropout at endline by arm** \| Control \| PM+ \| Total \| \| --- \| --- \| --- \| --- \| \|  \| (N = 1) \| (N = 2) \| (N = 3) \| |
| \|  \|  \|  \|  \| \| --- \| --- \| --- \| --- \| \| Death \| 1 (100.0%) \| 1 (50.0%) \| 2 (66.7%) \| \| Physical sickness \| 0 (0.0%) \| 1 (50.0%) \| 1 (33.3%) \| |

**Table B. Cluster size by arm (i.e. of the ITT population)**

| \|  \| Control \| PM+ \| Total \| \| --- \| --- \| --- \| --- \| \|  \| (N = 36) \| (N = 36) \| (N = 72) \| |
| --- | --- | --- | --- | --- | --- | --- | --- | --- |
| \| **Baseline overall** \|  \|  \|  \| \| --- \| --- \| --- \| --- \| \| Mean (SD) \| 8.5 (0.9) \| 8.5 (1.2) \| 8.5 (1.0) \| \| Median (Q1, Q3) \| 8 (8, 9) \| 8 (8, 9) \| 8 (8, 9) \| \| Min, Max \| 7, 11 \| 5, 10 \| 5, 11 \| \| **Baseline female** \|  \|  \|  \| \| Mean (SD) \| 8.7 (0.9) \| 8.7 (1.1) \| 8.7 (1.0) \| \| Median (Q1, Q3) \| 9 (8, 9) \| 9 (8, 9) \| 9 (8, 9) \| \| Min, Max \| 7, 11 \| 5, 10 \| 5, 11 \| \| **Baseline male** \|  \|  \|  \| \| Mean (SD) \| 7.9 (0.7) \| 7.7 (1.4) \| 7.8 (1.1) \| \| Median (Q1, Q3) \| 8 (7, 8) \| 8 (7, 9) \| 8 (7, 8) \| \| Min, Max \| 7, 9 \| 5, 9 \| 5, 9 \| \| **Midline overall** \|  \|  \|  \| \| Mean (SD) \| 8.4 (1.0) \| 8.4 (1.2) \| 8.4 (1.1) \| \| Median (Q1, Q3) \| 8 (8, 9) \| 8 (8, 9) \| 8 (8, 9) \| \| Min, Max \| 7, 11 \| 5, 10 \| 5, 11 \| \| **Midline female** \|  \|  \|  \| \| Mean (SD) \| 8.6 (0.9) \| 8.6 (1.1) \| 8.6 (1.0) \| \| Median (Q1, Q3) \| 8 (8, 9) \| 8 (8, 9) \| 8 (8, 9) \| \| Min, Max \| 7, 11 \| 5, 10 \| 5, 11 \| \| **Midline male** \|  \|  \|  \| \| Mean (SD) \| 7.4 (0.5) \| 7.6 (1.4) \| 7.5 (1.0) \| \| Median (Q1, Q3) \| 7 (7, 8) \| 8 (7, 9) \| 8 (7, 8) \| \| Min, Max \| 7, 8 \| 5, 9 \| 5, 9 \| \| **Endline overall** \|  \|  \|  \| \| Mean (SD) \| 8.4 (1.0) \| 8.4 (1.2) \| 8.4 (1.1) \| \| Median (Q1, Q3) \| 8 (8, 9) \| 8 (8, 9) \| 8 (8, 9) \| \| Min, Max \| 7, 11 \| 5, 10 \| 5, 11 \| \| **Endline female** \|  \|  \|  \| \| Mean (SD) \| 8.6 (0.9) \| 8.6 (1.1) \| 8.6 (1.0) \| \| Median (Q1, Q3) \| 8 (8, 9) \| 8 (8, 9) \| 8 (8, 9) \| \| Min, Max \| 7, 11 \| 5, 10 \| 5, 11 \| \| **Endline male** \|  \|  \|  \| \| Mean (SD) \| 7.6 (0.8) \| 7.4 (1.4) \| 7.5 (1.1) \| \| Median (Q1, Q3) \| 7 (7, 8) \| 7 (7, 9) \| 7 (7, 8) \| \| Min, Max \| 7, 9 \| 5, 9 \| 5, 9 \| |

**Table C. Baseline characteristics by gender and arm (i.e. of the ITT population)**

| \|  \| Female in Control \| Female in PM+ \| Male in Control \| Male in PM+ \| Total \| \| --- \| --- \| --- \| --- \| --- \| --- \| \|  \| (N = 251) \| (N = 251) \| (N = 55) \| (N = 54) \| (N = 611) \| |
| --- | --- | --- | --- | --- | --- | --- | --- | --- | --- | --- | --- | --- |
| \| **Age (years)** \|  \|  \|  \|  \|  \| \| --- \| --- \| --- \| --- \| --- \| --- \| \| Mean (SD) \| 43.5 (13.3) \| 43.6 (13.6) \| 46.9 (16.4) \| 54.0 (17.1) \| 44.8 (14.4) \| \| Median (Q1, Q3) \| 45.0 (33.0, 53.0) \| 42.0 (34.0, 52.0) \| 50.0 (33.0, 58.0) \| 53.5 (38.0, 65.0) \| 45.0 (34.0, 55.0) \| \| Min, Max \| 18.0, 82.0 \| 18.0, 91.0 \| 18.0, 83.0 \| 21.0, 85.0 \| 18.0, 91.0 \| \| **Age categories** \|  \|  \|  \|  \|  \| \| < 30 \| 41 (16.3%) \| 35 (13.9%) \| 9 (16.4%) \| 3 (5.6%) \| 88 (14.4%) \| \| 30 - < 40 \| 58 (23.1%) \| 67 (26.7%) \| 11 (20.0%) \| 11 (20.4%) \| 147 (24.1%) \| \| 40 - < 50 \| 72 (28.7%) \| 69 (27.5%) \| 7 (12.7%) \| 3 (5.6%) \| 151 (24.7%) \| \| 50 - < 60 \| 44 (17.5%) \| 42 (16.7%) \| 18 (32.7%) \| 15 (27.8%) \| 119 (19.5%) \| \| 60 - <70 \| 28 (11.2%) \| 26 (10.4%) \| 6 (10.9%) \| 12 (22.2%) \| 72 (11.8%) \| \| 70+ \| 8 (3.2%) \| 12 (4.8%) \| 4 (7.3%) \| 10 (18.5%) \| 34 (5.6%) \| \| **Gender** \|  \|  \|  \|  \|  \| \| Male \| 0 (0.0%) \| 0 (0.0%) \| 55 (100.0%) \| 54 (100.0%) \| 109 (17.8%) \| \| Female \| 251 (100.0%) \| 251 (100.0%) \| 0 (0.0%) \| 0 (0.0%) \| 502 (82.2%) \| \| **Education** \|  \|  \|  \|  \|  \| \| Cant read/write \| 78 (31.1%) \| 77 (30.7%) \| 10 (18.2%) \| 11 (20.4%) \| 176 (28.8%) \| \| Literate or informal education \| 72 (28.7%) \| 70 (27.9%) \| 12 (21.8%) \| 10 (18.5%) \| 164 (26.8%) \| \| Primary level \| 54 (21.5%) \| 60 (23.9%) \| 18 (32.7%) \| 17 (31.5%) \| 149 (24.4%) \| \| Secondary \| 34 (13.5%) \| 31 (12.4%) \| 13 (23.6%) \| 14 (25.9%) \| 92 (15.1%) \| \| Higher secondary \| 11 (4.4%) \| 12 (4.8%) \| 2 (3.6%) \| 1 (1.9%) \| 26 (4.3%) \| \| University \| 2 (0.8%) \| 1 (0.4%) \| 0 (0.0%) \| 1 (1.9%) \| 4 (0.7%) \| \| **Occupation** \|  \|  \|  \|  \|  \| \| Farmer \| 75 (29.9%) \| 66 (26.3%) \| 29 (52.7%) \| 27 (50.0%) \| 197 (32.2%) \| \| Business/job \| 11 (4.3%) \| 25 (9.9%) \| 12 (21.8%) \| 9 (16.6%) \| 67 (10.9%) \| \| Daily wage laborer \| 28 (11.2%) \| 28 (11.2%) \| 7 (12.7%) \| 5 (9.3%) \| 68 (11.1%) \| \| Unemployed \| 8 (3.2%) \| 4 (1.6%) \| 1 (1.8%) \| 10 (18.5%) \| 23 (3.8%) \| \| Student \| 5 (2.0%) \| 4 (1.6%) \| 3 (5.5%) \| 0 (0.0%) \| 12 (2.0%) \| \| Housewife \| 113 (45.0%) \| 120 (47.8%) \| 0 (0.0%) \| 0 (0.0%) \| 233 (38.1%) \| \| Other \| 1 (0.4%) \| 4 (1.6%) \| 3 (5.5%) \| 3 (5.6%) \| 11 (1.8%) \| \| **Caste** \|  \|  \|  \|  \|  \| \| Brahmin/Chetri \| 86 (34.2%) \| 86 (34.2%) \| 24 (43.6%) \| 24 (44.4%) \| 220 (36.0%) \| \| Local Indigenous and Madhesi \| 48 (19.1%) \| 52 (20.7%) \| 0 (0.0%) \| 5 (9.3%) \| 99 (16.2%) \| \| Janjati \| 58 (23.1%) \| 61 (24.3%) \| 20 (36.4%) \| 12 (22.2%) \| 151 (24.7%) \| \| Other \| 59 (23.5%) \| 52 (20.7%) \| 11 (20.0%) \| 13 (24.1%) \| 135 (22.1%) \| \| **Religion** \|  \|  \|  \|  \|  \| \| Hindu \| 215 (85.7%) \| 217 (86.5%) \| 42 (76.4%) \| 50 (92.6%) \| 524 (85.8%) \| \| Buddhist \| 4 (1.6%) \| 8 (3.2%) \| 6 (10.9%) \| 0 (0.0%) \| 18 (2.9%) \| \| Muslim \| 1 (0.4%) \| 0 (0.0%) \| 0 (0.0%) \| 0 (0.0%) \| 1 (0.2%) \| \| Christian \| 16 (6.4%) \| 16 (6.4%) \| 3 (5.5%) \| 1 (1.9%) \| 36 (5.9%) \| \| No religion \| 1 (0.4%) \| 1 (0.4%) \| 0 (0.0%) \| 0 (0.0%) \| 2 (0.3%) \| \| Other \| 14 (5.6%) \| 9 (3.6%) \| 4 (7.3%) \| 3 (5.6%) \| 30 (4.9%) \| \| **Martial status** \|  \|  \|  \|  \|  \| \| Unmarried \| 8 (3.2%) \| 12 (4.8%) \| 8 (14.5%) \| 5 (9.3%) \| 33 (5.4%) \| \| Married \| 204 (81.3%) \| 200 (79.7%) \| 45 (81.8%) \| 42 (77.8%) \| 491 (80.4%) \| \| Widowed \| 26 (10.4%) \| 36 (14.3%) \| 1 (1.8%) \| 3 (5.6%) \| 66 (10.8%) \| \| Divorced \| 3 (1.2%) \| 1 (0.4%) \| 0 (0.0%) \| 2 (3.7%) \| 6 (1.0%) \| \| Separated \| 10 (4.0%) \| 2 (0.8%) \| 1 (1.8%) \| 2 (3.7%) \| 15 (2.5%) \| \| **Most used language** \|  \|  \|  \|  \|  \| \| Nepali \| 197 (78.5%) \| 198 (78.9%) \| 55 (100.0%) \| 51 (94.4%) \| 501 (82.0%) \| \| Maithali \| 8 (3.2%) \| 3 (1.2%) \| 0 (0.0%) \| 0 (0.0%) \| 11 (1.8%) \| \| Tharu \| 27 (10.8%) \| 36 (14.3%) \| 0 (0.0%) \| 0 (0.0%) \| 63 (10.3%) \| \| Rajbansi or Dhimal \| 6 (2.3%) \| 1 (0.3%) \| 0 (0.0%) \| 2 (3.7%) \| 9 (1.4%) \| \| Rai,Limbu,Tamang \| 5 (1.9%) \| 0 (0.0%) \| 0 (0.0%) \| 1 (1.8%) \| 5 (0.8%) \| \| Other \| 8 (3.2%) \| 13 (5.2%) \| 0 (0.0%) \| 1 (1.9%) \| 22 (3.6%) \| \| **Number of members in household** \|  \|  \|  \|  \|  \| \| Living alone \| 11 (4.4%) \| 9 (3.6%) \| 0 (0.0%) \| 0 (0.0%) \| 20 (3.3%) \| \| 1 other person \| 26 (10.4%) \| 23 (9.2%) \| 3 (5.5%) \| 6 (11.1%) \| 58 (9.5%) \| \| 2 – 3 other people \| 95 (37.8%) \| 106 (42.2%) \| 24 (43.6%) \| 16 (29.6%) \| 247 (40.4%) \| \| 4 or more others \| 113 (45.0%) \| 113 (45.0%) \| 28 (50.9%) \| 32 (59.2%) \| 286 (46.8%) \| \| **Chronic diseases** \|  \|  \|  \|  \|  \| \| No \| 179 (71.3%) \| 178 (70.9%) \| 33 (60.0%) \| 31 (57.4%) \| 421 (68.9%) \| \| Yes \| 72 (28.7%) \| 73 (29.1%) \| 22 (40.0%) \| 23 (42.6%) \| 190 (31.1%) \| \| **If yes to chronic disease** \|  \|  \|  \|  \|  \| \| Cancer \| 2 (2.8%) \| 2 (2.7%) \| 0 (0.0%) \| 1 (4.3%) \| 5 (2.6%) \| \| Diabetes \| 13 (18.1%) \| 11 (15.1%) \| 4 (18.2%) \| 7 (30.4%) \| 35 (18.4%) \| \| Hypertension \| 28 (38.9%) \| 30 (41.1%) \| 8 (36.4%) \| 6 (26.1%) \| 72 (37.9%) \| \| Asthma \| 11 (15.3%) \| 14 (19.2%) \| 3 (13.6%) \| 7 (30.4%) \| 35 (18.4%) \| \| Other \| 18 (25.0%) \| 16 (21.9%) \| 7 (31.8%) \| 2 (8.7%) \| 43 (22.6%) \| \| **Who do you live with** \|  \|  \|  \|  \|  \| \| Extended family with spouse \| 80 (31.9%) \| 58 (23.1%) \| 21 (38.2%) \| 23 (42.6%) \| 182 (29.8%) \| \| Extended family without spouse \| 13 (5.2%) \| 18 (7.2%) \| 2 (3.6%) \| 5 (9.3%) \| 38 (6.2%) \| \| With parents \| 4 (1.6%) \| 5 (2.0%) \| 6 (10.9%) \| 4 (7.4%) \| 19 (3.1%) \| \| Maternal home \| 6 (2.4%) \| 5 (2.0%) \| 0 (0.0%) \| 0 (0.0%) \| 11 (1.8%) \| \| Spouse only \| 18 (7.2%) \| 12 (4.8%) \| 2 (3.6%) \| 5 (9.3%) \| 37 (6.1%) \| \| Spouse and children only \| 87 (34.7%) \| 106 (42.2%) \| 20 (36.4%) \| 10 (18.5%) \| 223 (36.5%) \| \| Other \| 43 (17.1%) \| 47 (18.7%) \| 4 (7.3%) \| 7 (13.0%) \| 101 (16.5%) \| \| **Concrete Building** \|  \|  \|  \|  \|  \| \| No \| 220 (87.6%) \| 212 (84.5%) \| 47 (85.5%) \| 45 (83.3%) \| 524 (85.8%) \| \| Yes \| 31 (12.4%) \| 39 (15.5%) \| 8 (14.5%) \| 9 (16.7%) \| 87 (14.2%) \| \| **Electricity** \|  \|  \|  \|  \|  \| \| No \| 34 (13.5%) \| 28 (11.2%) \| 1 (1.8%) \| 0 (0.0%) \| 63 (10.3%) \| \| Yes \| 217 (86.5%) \| 223 (88.8%) \| 54 (98.2%) \| 54 (100.0%) \| 548 (89.7%) \| \| **Drinking water** \|  \|  \|  \|  \|  \| \| No \| 26 (10.4%) \| 33 (13.1%) \| 4 (7.3%) \| 2 (3.7%) \| 65 (10.6%) \| \| Yes \| 225 (89.6%) \| 218 (86.9%) \| 51 (92.7%) \| 52 (96.3%) \| 546 (89.4%) \| \| **Radio** \|  \|  \|  \|  \|  \| \| No \| 184 (73.3%) \| 193 (76.9%) \| 37 (67.3%) \| 34 (63.0%) \| 448 (73.3%) \| \| Yes \| 67 (26.7%) \| 58 (23.1%) \| 18 (32.7%) \| 20 (37.0%) \| 163 (26.7%) \| \| **Television** \|  \|  \|  \|  \|  \| \| No \| 101 (40.2%) \| 92 (36.7%) \| 19 (34.5%) \| 23 (42.6%) \| 235 (38.5%) \| \| Yes \| 150 (59.8%) \| 159 (63.3%) \| 36 (65.5%) \| 31 (57.4%) \| 376 (61.5%) \| \| **Simple mobile phone** \|  \|  \|  \|  \|  \| \| No \| 50 (19.9%) \| 55 (21.9%) \| 4 (7.3%) \| 8 (14.8%) \| 117 (19.1%) \| \| Yes \| 201 (80.1%) \| 196 (78.1%) \| 51 (92.7%) \| 46 (85.2%) \| 494 (80.9%) \| \| **Smart mobile phone** \|  \|  \|  \|  \|  \| \| No \| 137 (54.6%) \| 113 (46.1%) \| 18 (32.7%) \| 23 (42.6%) \| 291 (48.1%) \| \| Yes \| 114 (45.4%) \| 132 (53.9%) \| 37 (67.3%) \| 31 (57.4%) \| 314 (51.9%) \| \| **Cycle** \|  \|  \|  \|  \|  \| \| No \| 90 (35.9%) \| 63 (25.1%) \| 14 (25.5%) \| 16 (29.6%) \| 183 (30.0%) \| \| Yes \| 161 (64.1%) \| 188 (74.9%) \| 41 (74.5%) \| 38 (70.4%) \| 428 (70.0%) \| \| **LP Gas** \|  \|  \|  \|  \|  \| \| No \| 75 (29.9%) \| 64 (25.5%) \| 7 (12.7%) \| 10 (18.5%) \| 156 (25.5%) \| \| Yes \| 176 (70.1%) \| 187 (74.5%) \| 48 (87.3%) \| 44 (81.5%) \| 455 (74.5%) \| |

**Table D. Baseline characteristics by arm of the completers population**

| \|  \| Control \| PM+ Completer \| PM+ Non-Completer \| Total \| \| --- \| --- \| --- \| --- \| --- \| \|  \| (N = 306) \| (N = 238) \| (N = 67) \| (N = 611) \| |
| --- | --- | --- | --- | --- | --- | --- | --- | --- | --- | --- |
| \| **Age (years)** \|  \|  \|  \|  \| \| --- \| --- \| --- \| --- \| --- \| \| Mean (SD) \| 44.1 (14.0) \| 46.5 (15.0) \| 41.7 (13.6) \| 44.8 (14.4) \| \| Median (Q1, Q3) \| 45.0 (33.0, 54.0) \| 45.0 (35.0, 58.0) \| 40.0 (30.0, 50.0) \| 45.0 (34.0, 55.0) \| \| Min, Max \| 18.0, 83.0 \| 18.0, 91.0 \| 21.0, 81.0 \| 18.0, 91.0 \| \| **Age categories (years)** \|  \|  \|  \|  \| \| < 30 \| 50 (16.3%) \| 24 (10.1%) \| 14 (20.9%) \| 88 (14.4%) \| \| 30 - < 40 \| 69 (22.5%) \| 61 (25.6%) \| 17 (25.4%) \| 147 (24.1%) \| \| 40 - < 50 \| 79 (25.8%) \| 56 (23.5%) \| 16 (23.9%) \| 151 (24.7%) \| \| 50 - < 60 \| 62 (20.3%) \| 43 (18.1%) \| 14 (20.9%) \| 119 (19.5%) \| \| 60 - <70 \| 34 (11.1%) \| 34 (14.3%) \| 4 (6.0%) \| 72 (11.8%) \| \| 70+ \| 12 (3.9%) \| 20 (8.4%) \| 2 (3.0%) \| 34 (5.6%) \| \| **Gender** \|  \|  \|  \|  \| \| Male \| 55 (18.0%) \| 35 (14.7%) \| 19 (28.4%) \| 109 (17.8%) \| \| Female \| 251 (82.0%) \| 203 (85.3%) \| 48 (71.6%) \| 502 (82.2%) \| \| **Education** \|  \|  \|  \|  \| \| Cant read or write \| 88 (28.8%) \| 70 (29.4%) \| 18 (26.9%) \| 176 (28.8%) \| \| Literate or informal education \| 84 (27.5%) \| 70 (29.4%) \| 10 (14.9%) \| 164 (26.8%) \| \| Primary level \| 72 (23.5%) \| 56 (23.5%) \| 21 (31.3%) \| 149 (24.4%) \| \| Secondary \| 47 (15.4%) \| 29 (12.2%) \| 16 (23.9%) \| 92 (15.1%) \| \| Higher secondary \| 13 (4.2%) \| 11 (4.6%) \| 2 (3.0%) \| 26 (4.3%) \| \| University \| 2 (0.7%) \| 2 (0.8%) \| 0 (0.0%) \| 4 (0.7%) \| \| **Occupation** \|  \|  \|  \|  \| \| Farmer \| 104 (34.0%) \| 75 (31.5%) \| 18 (26.9%) \| 197 (32.2%) \| \| Business or Job \| 33 (10.7%) \| 22 (9.2%) \| 12 (17.9%) \| 67 (10.9%) \| \| Daily wage laborer \| 35 (11.4%) \| 23 (9.7%) \| 10 (14.9%) \| 68 (11.1%) \| \| Unemployed \| 9 (2.9%) \| 11 (4.6%) \| 3 (4.5%) \| 23 (3.8%) \| \| Student \| 8 (2.6%) \| 4 (1.7%) \| 0 (0.0%) \| 12 (2.0%) \| \| Housewife \| 113 (36.9%) \| 100 (42.0%) \| 20 (29.9%) \| 233 (38.1%) \| \| Other \| 4 (1.3%) \| 3 (1.3%) \| 4 (6.0%) \| 11 (1.8%) \| \| **Caste** \|  \|  \|  \|  \| \| Brahmin/Chetri \| 110 (35.9%) \| 88 (36.9%) \| 22 (32.8%) \| 220 (36.0%) \| \| Local Indigenous and Madhesi \| 48 (15.7%) \| 17.6 (12.6%) \| 15 (22.4%) \| 105 (17.2%) \| \| Janjati \| 78 (25.5%) \| 59 (24.8%) \| 14 (20.9%) \| 151 (24.7%) \| \| Other \| 70 (22.9%) \| 49 (20.6%) \| 16 (23.9%) \| 135 (22.1%) \| \| **Religion** \|  \|  \|  \|  \| \| Hindu \| 257 (84.0%) \| 208 (87.4%) \| 59 (88.1%) \| 524 (85.8%) \| \| Buddhist \| 10 (3.3%) \| 6 (2.5%) \| 2 (3.0%) \| 18 (2.9%) \| \| Muslim \| 1 (0.3%) \| 0 (0.0%) \| 0 (0.0%) \| 1 (0.2%) \| \| Christian \| 19 (6.2%) \| 16 (6.7%) \| 1 (1.5%) \| 36 (5.9%) \| \| No religion \| 1 (0.3%) \| 1 (0.4%) \| 0 (0.0%) \| 2 (0.3%) \| \| Other \| 18 (5.9%) \| 7 (2.9%) \| 5 (7.5%) \| 30 (4.9%) \| \| **Martial status** \|  \|  \|  \|  \| \| Unmarried \| 16 (5.2%) \| 12 (5.0%) \| 5 (7.5%) \| 33 (5.4%) \| \| Married \| 249 (81.4%) \| 188 (79.0%) \| 54 (80.6%) \| 491 (80.4%) \| \| Widowed \| 27 (8.8%) \| 33 (13.9%) \| 6 (9.0%) \| 66 (10.8%) \| \| Divorced \| 3 (1.0%) \| 1 (0.4%) \| 2 (3.0%) \| 6 (1.0%) \| \| Separated \| 11 (3.6%) \| 4 (1.7%) \| 0 (0.0%) \| 15 (2.5%) \| \| **Most used language** \|  \|  \|  \|  \| \| Nepali \| 252 (82.4%) \| 193 (81.1%) \| 56 (83.6%) \| 501 (82.0%) \| \| Maithali \| 8 (2.6%) \| 3 (1.3%) \| 0 (0.0%) \| 11 (1.8%) \| \| Tharu \| 27 (8.8%) \| 31 (13.0%) \| 5 (7.5%) \| 63 (10.3%) \| \| Rajbansi or Dhimal \| 6 (1.9%) \| 1 (0.4%) \| 2 (2.9%) \| 9 (1.4%) \| \| Rai/Limbu or Tamang \| 5 (1.6%) \| 0 (0.0%) \| 0 (0.0%) \| 5 (0.8%) \| \| Other \| 8 (2.6%) \| 10 (4.2%) \| 4 (6.0%) \| 22 (3.6%) \| \| **Number of members in household** \|  \|  \|  \|  \| \| Living alone \| 11 (3.6%) \| 9 (3.8%) \| 0 (0.0%) \| 20 (3.3%) \| \| With 1 other person \| 29 (9.5%) \| 22 (9.2%) \| 7 (10.4%) \| 58 (9.5%) \| \| With 2 – 3 other people \| 125 (13.7%) \| 92 (13.4%) \| 30 (28.4%) \| 247 (15.2%) \| \| With 4 or more people \| 141 (40.8%) \| 115 (28.3%) \| 30 (44.7%) \| 286 (46.8%) \| \| **Chronic diseases** \|  \|  \|  \|  \| \| No \| 212 (69.3%) \| 172 (72.3%) \| 37 (55.2%) \| 421 (68.9%) \| \| Yes \| 94 (30.7%) \| 66 (27.7%) \| 30 (44.8%) \| 190 (31.1%) \| \| **If yes to chronic disease** \|  \|  \|  \|  \| \| Cancer \| 2 (2.1%) \| 1 (1.5%) \| 2 (6.7%) \| 5 (2.6%) \| \| Diabetes \| 17 (18.1%) \| 9 (13.6%) \| 9 (30.0%) \| 35 (18.4%) \| \| Hypertension \| 36 (38.3%) \| 28 (42.4%) \| 8 (26.7%) \| 72 (37.9%) \| \| Asthma \| 14 (14.9%) \| 18 (27.3%) \| 3 (10.0%) \| 35 (18.4%) \| \| Other \| 25 (26.6%) \| 10 (15.2%) \| 8 (26.7%) \| 43 (22.6%) \| \| **Who do you live with** \|  \|  \|  \|  \| \| Extended family with spouse \| 101 (33.0%) \| 60 (25.2%) \| 21 (31.3%) \| 182 (29.8%) \| \| Extended family without spouse \| 15 (4.9%) \| 21 (8.8%) \| 2 (3.0%) \| 38 (6.2%) \| \| With parents \| 10 (3.3%) \| 7 (2.9%) \| 2 (3.0%) \| 19 (3.1%) \| \| Maternal home *(maiti*) \| 6 (2.0%) \| 4 (1.7%) \| 1 (1.5%) \| 11 (1.8%) \| \| Spouse only \| 20 (6.5%) \| 13 (5.5%) \| 4 (6.0%) \| 37 (6.1%) \| \| Spouse and children only \| 107 (35.0%) \| 89 (37.4%) \| 27 (40.3%) \| 223 (36.5%) \| \| Other \| 47 (15.4%) \| 44 (18.5%) \| 10 (14.9%) \| 101 (16.5%) \| \| **Concrete Building** \|  \|  \|  \|  \| \| No \| 267 (87.3%) \| 201 (84.5%) \| 56 (83.6%) \| 524 (85.8%) \| \| Yes \| 39 (12.7%) \| 37 (15.5%) \| 11 (16.4%) \| 87 (14.2%) \| \| **Electricity** \|  \|  \|  \|  \| \| No \| 35 (11.4%) \| 22 (9.2%) \| 6 (9.0%) \| 63 (10.3%) \| \| Yes \| 271 (88.6%) \| 216 (90.8%) \| 61 (91.0%) \| 548 (89.7%) \| \| **Drinking water** \|  \|  \|  \|  \| \| No \| 30 (9.8%) \| 29 (12.2%) \| 6 (9.0%) \| 65 (10.6%) \| \| Yes \| 276 (90.2%) \| 209 (87.8%) \| 61 (91.0%) \| 546 (89.4%) \| \| **Radio** \|  \|  \|  \|  \| \| No \| 221 (72.2%) \| 183 (76.9%) \| 44 (65.7%) \| 448 (73.3%) \| \| Yes \| 85 (27.8%) \| 55 (23.1%) \| 23 (34.3%) \| 163 (26.7%) \| \| **Television** \|  \|  \|  \|  \| \| No \| 120 (39.2%) \| 92 (38.7%) \| 23 (34.3%) \| 235 (38.5%) \| \| Yes \| 186 (60.8%) \| 146 (61.3%) \| 44 (65.7%) \| 376 (61.5%) \| \| **Simple mobile phone** \|  \|  \|  \|  \| \| No \| 54 (17.6%) \| 46 (19.3%) \| 17 (25.4%) \| 117 (19.1%) \| \| Yes \| 252 (82.4%) \| 192 (80.7%) \| 50 (74.6%) \| 494 (80.9%) \| \| **Smart mobile phone** \|  \|  \|  \|  \| \| No \| 155 (50.7%) \| 105 (44.9%) \| 31 (47.7%) \| 291 (48.1%) \| \| Yes \| 151 (49.3%) \| 129 (55.1%) \| 34 (52.3%) \| 314 (51.9%) \| \| **Cycle** \|  \|  \|  \|  \| \| No \| 104 (34.0%) \| 61 (25.6%) \| 18 (26.9%) \| 183 (30.0%) \| \| Yes \| 202 (66.0%) \| 177 (74.4%) \| 49 (73.1%) \| 428 (70.0%) \| \| **LP Gas** \|  \|  \|  \|  \| \| No \| 82 (26.8%) \| 63 (26.5%) \| 11 (16.4%) \| 156 (25.5%) \| \| Yes \| 224 (73.2%) \| 175 (73.5%) \| 56 (83.6%) \| 455 (74.5%) \| |

**Table E: Summary of fidelity checklist (FC) score by session**^a^

|  | n^b^ | Mean | SD | Median | Min | Max |
| --- | --- | --- | --- | --- | --- | --- |
|  |  |  |  |  |  |  |
| Session 1 | 18 | 2.8 | 0.2 | 2.8 | 2.4 | 3.0 |
| Session 2 | 13 | 2.8 | 0.1 | 2.8 | 2.4 | 3.0 |
| Session 3 | 11 | 2.9 | 0.2 | 3.0 | 2.6 | 3.0 |
| Session 4 | 15 | 2.7 | 0.1 | 2.8 | 2.4 | 2.9 |
| Session 5 | 20 | 2.8 | 0.1 | 2.9 | 2.7 | 3.0 |

^a^ Average values of FC are computed for each session of each ward, and then summarized here for each session across wards.

^b^ 79 sessions total were evaluated in-person by the Clinical Supervisors. Clinical Supervisors evaluated at least 2 sessions per PM+ group. Clinical Supervisors attempted to evaluate at least one of each session (session 1, 2, 3, 4, and 5) for each facilitator. However, facilitators were evaluated during different sessions depending on which sessions they needed the most support on and the time schedule of the Clinical Supervisor. FC data is unavailable for session 5 of ward S9 and session 5 of ward P1.

**Table F. Intervention participation**

| \|  \| Total \| \| --- \| --- \| \|  \| (N = 305) \| |
| --- | --- | --- | --- | --- |
| \| **Number of sessions attended** \|  \| \| --- \| --- \| \| 0 \| 15 (4.9%) \| \| 1 \| 5 (1.6%) \| \| 2 \| 21 (6.9%) \| \| 3 \| 26 (8.5%) \| \| 4 \| 72 (23.6%) \| \| 5 \| 166 (54.4%) \| \| **Completer (attended 4 or 5 sessions)** \|  \| \| No \| 67 (22.0%) \| \| Yes \| 238 (78.0%) \| \| **Session 1** \|  \| \| No \| 40 (13.1%) \| \| Yes \| 265 (86.9%) \| \| **Session 2** \|  \| \| No \| 59 (19.3%) \| \| Yes \| 246 (80.7%) \| \| **Session 3** \|  \| \| No \| 67 (22.0%) \| \| Yes \| 238 (78.0%) \| \| **Session 4** \|  \| \| No \| 60 (19.7%) \| \| Yes \| 245 (80.3%) \| \| **Session 5** \|  \| \| No \| 56 (18.4%) \| \| Yes \| 249 (81.6%) \| |

**Table G. Service use by arm (i.e. of the ITT population)**

| \|  \| Control \| PM+ Control \| \| --- \| --- \| --- \| \|  \| (N = 306) \| (N = 305) (N = 611) \| |
| --- | --- | --- | --- | --- | --- | --- |
| \| **Medication use for mental health problems since start of study** \|  \|  \|  \| \| --- \| --- \| --- \| --- \| \| No \| 288 (94.1%) \| 290 (95.0%) 578 (95.6%) \|  \| \| Yes \| 9 (2.9%) \| 9 (2.9%) 18 (2.9%) \|  \| \| Do not know \| 9 (2.9%) \| 6 (1.9%) 15 (2.4%) \|  \| \| **Received counseling services (i.e. with a counselor, doctor, religious advisor) since start of study** \|  \|  \|  \| \| One time \| 5 (1.6%) \| 1 (0.3%) 6 (1.0%) \|  \| \| 2 – 4 times \| 2 (0.6%) \| 1 (0.3%) 3 (0.5%) \|  \| \| Did not receive any counseling outside of study \| 299 (97.7%) \| 303 (99.3%) 602 (98.5%) \|  \| \| **Received traditional healing since start of the study** \|  \|  \|  \| \| 0 times \| 271 (88.5%) \| 272 (89.2%) 543 (88.9%) \|  \| \| 1 – 4 times \| 33 (10.7%) \| 30 (9.8%) 63 (10.3%) \|  \| \| 5 – 10 times \| 2 (0.7%) \| 2 (0.7%) 4 (0.7%) \|  \| \| >10 times \| 0 (0.0%) \| 1 (0.3%) 1 (0.2%) \|  \| \| **Ever taken medication for mental health problems** \|  \|  \|  \| \| No \| 263 (85.9%) \| 287 (94.1%) \|  \| \| Yes \| 31 (10.1%) \| 10 (3.3%) \|  \| \| Do not know \| 12 (3.9%) \| 8 (2.6%) \|  \| \| **Ever received counseling services (i.e. counselor, doctor, religious advisor etc) before start of study** \|  \|  \|  \| \| 0 times \| 291 (95.1%) \| 297 (97.4%) \|  \| \| 1 – 4 times \| 7 (2.3%) \| 2 (0.7%) \|  \| \| 5 – 10 times \| 6 (1.9%) \| 4 (1.3%) \|  \| \| >10 times \| 2 (0.7%) \| 2 (0.7%) \|  \| |

**Table H. Outcome summaries by arm (i.e. of the ITT population)**

| \|  \| Control \| PM+ \| Total \| \| --- \| --- \| --- \| --- \| \|  \| (N = 306) \| (N = 305) \| (N = 611) \| |
| --- | --- | --- | --- | --- | --- | --- | --- | --- |
| \| **GHQ-12 total (0-36)** \|  \|  \|  \| \| --- \| --- \| --- \| --- \| \| Baseline \|  \|  \|  \| \| Mean (SD) \| 20.9 (6.0) \| 21.2 (6.3) \| 21.0 (6.1) \| \| Median (Q1, Q3) \| 21.0 (17.0, 25.0) \| 21.0 (17.0, 26.0) \| 21.0 (17.0, 25.0) \| \| Min, Max \| 0.0, 35.0 \| 4.0, 35.0 \| 0.0, 35.0 \| \| N (% Non-missing) \| 306 (100.0%) \| 305 (100.0%) \| 611 (100.0%) \| \| Midline \|  \|  \|  \| \| Mean (SD) \| 20.3 (6.6) \| 17.7 (6.9) \| 19.0 (6.9) \| \| Median (Q1, Q3) \| 20.0 (17.0, 25.0) \| 17.0 (13.0, 22.0) \| 19.0 (14.0, 23.0) \| \| Min, Max \| 2.0, 36.0 \| 1.0, 35.0 \| 1.0, 36.0 \| \| N (% Non-missing) \| 301 (98.4%) \| 303 (99.3%) \| 604 (98.9%) \| \| Endline \|  \|  \|  \| \| Mean (SD) \| 19.3 (6.5) \| 18.1 (7.0) \| 18.7 (6.7) \| \| Median (Q1, Q3) \| 20.0 (15.0, 24.0) \| 18.0 (13.0, 22.0) \| 19.0 (14.0, 23.0) \| \| Min, Max \| 1.0, 35.0 \| 0.0, 36.0 \| 0.0, 36.0 \| \| N (% Non-missing) \| 301 (98.4%) \| 301 (98.7%) \| 602 (98.5%) \| \| **WHODAS (items 1-12) total (0-48)** \|  \|  \|  \| \| Baseline \|  \|  \|  \| \| Mean (SD) \| 24.6 (6.5) \| 26.0 (6.8) \| 25.3 (6.7) \| \| Median (Q1, Q3) \| 23.0 (19.0, 28.0) \| 25.0 (20.0, 30.0) \| 24.0 (20.0, 29.0) \| \| Min, Max \| 17.0, 48.0 \| 17.0, 46.0 \| 17.0, 48.0 \| \| N (% Non-missing) \| 306 (100.0%) \| 305 (100.0%) \| 611 (100.0%) \| \| Midline \|  \|  \|  \| \| Mean (SD) \| 19.2 (9.6) \| 17.1 (8.8) \| 18.2 (9.3) \| \| Median (Q1, Q3) \| 19.0 (13.0, 26.0) \| 17.0 (10.0, 24.0) \| 18.0 (11.0, 24.0) \| \| Min, Max \| 0.0, 47.0 \| 0.0, 45.0 \| 0.0, 47.0 \| \| N (% Non-missing) \| 301 (98.4%) \| 303 (99.3%) \| 604 (98.9%) \| \| Endline \|  \|  \|  \| \| Mean (SD) \| 16.5 (8.6) \| 16.0 (9.6) \| 16.2 (9.1) \| \| Median (Q1, Q3) \| 16.0 (10.0, 22.0) \| 16.0 (8.0, 23.0) \| 16.0 (9.0, 23.0) \| \| Min, Max \| 0.0, 45.0 \| 0.0, 45.0 \| 0.0, 45.0 \| \| N (% Non-missing) \| 301 (98.4%) \| 301 (98.7%) \| 602 (98.5%) \| \| **PHQ-9 (items 1-10) total (0-30)** \|  \|  \|  \| \| Baseline \|  \|  \|  \| \| Mean (SD) \| 11.9 (5.0) \| 12.7 (4.9) \| 12.3 (5.0) \| \| Median (Q1, Q3) \| 12.0 (8.0, 15.0) \| 12.0 (9.0, 16.0) \| 12.0 (9.0, 16.0) \| \| Min, Max \| 0.0, 27.0 \| 1.0, 27.0 \| 0.0, 27.0 \| \| N (% Non-missing) \| 306 (100.0%) \| 305 (100.0%) \| 611 (100.0%) \| \| Midline \|  \|  \|  \| \| Mean (SD) \| 10.9 (5.2) \| 9.6 (5.0) \| 10.2 (5.1) \| \| Median (Q1, Q3) \| 11.0 (7.0, 14.0) \| 9.0 (6.0, 13.0) \| 10.0 (6.0, 13.0) \| \| Min, Max \| 0.0, 27.0 \| 0.0, 24.0 \| 0.0, 27.0 \| \| N (% Non-missing) \| 301 (98.4%) \| 303 (99.3%) \| 604 (98.9%) \| \| Endline \|  \|  \|  \| \| Mean (SD) \| 10.0 (4.4) \| 9.5 (5.2) \| 9.8 (4.8) \| \| Median (Q1, Q3) \| 10.0 (7.0, 13.0) \| 9.0 (6.0, 13.0) \| 10.0 (6.0, 13.0) \| \| Min, Max \| 0.0, 26.0 \| 0.0, 25.0 \| 0.0, 26.0 \| \| N (% Non-missing) \| 301 (98.4%) \| 301 (98.7%) \| 602 (98.5%) \| \| **PCL (items 1-8) total (8-40)** \|  \|  \|  \| \| Baseline \|  \|  \|  \| \| Mean (SD) \| 21.8 (7.0) \| 23.0 (6.8) \| 22.4 (6.9) \| \| Median (Q1, Q3) \| 21.5 (17.0, 26.0) \| 23.0 (18.0, 28.0) \| 22.0 (17.0, 27.0) \| \| Min, Max \| 8.0, 40.0 \| 8.0, 40.0 \| 8.0, 40.0 \| \| N (% Non-missing) \| 306 (100.0%) \| 305 (100.0%) \| 611 (100.0%) \| \| Midline \|  \|  \|  \| \| Mean (SD) \| 21.4 (6.9) \| 20.4 (6.9) \| 20.9 (6.9) \| \| Median (Q1, Q3) \| 21.0 (16.0, 26.0) \| 20.0 (15.0, 25.0) \| 20.5 (16.0, 26.0) \| \| Min, Max \| 8.0, 40.0 \| 8.0, 39.0 \| 8.0, 40.0 \| \| N (% Non-missing) \| 301 (98.4%) \| 303 (99.3%) \| 604 (98.9%) \| \| Endline \|  \|  \|  \| \| Mean (SD) \| 20.5 (6.6) \| 20.2 (7.1) \| 20.3 (6.9) \| \| Median (Q1, Q3) \| 21.0 (16.0, 25.0) \| 20.0 (15.0, 25.0) \| 20.0 (15.0, 25.0) \| \| Min, Max \| 8.0, 40.0 \| 8.0, 38.0 \| 8.0, 40.0 \| \| N (% Non-missing) \| 301 (98.4%) \| 301 (98.7%) \| 602 (98.5%) \| \| **MSPSS (items 1-12) total (12-60)** \|  \|  \|  \| \| Baseline \|  \|  \|  \| \| Mean (SD) \| 30.7 (9.5) \| 32.5 (9.9) \| 31.6 (9.7) \| \| Median (Q1, Q3) \| 31.0 (23.0, 37.0) \| 32.0 (26.0, 40.0) \| 32.0 (24.0, 38.0) \| \| Min, Max \| 12.0, 57.0 \| 12.0, 57.0 \| 12.0, 57.0 \| \| N (% Non-missing) \| 306 (100.0%) \| 305 (100.0%) \| 611 (100.0%) \| \| Midline \|  \|  \|  \| \| Mean (SD) \| 30.8 (9.5) \| 32.9 (8.7) \| 31.9 (9.1) \| \| Median (Q1, Q3) \| 31.0 (24.0, 38.0) \| 33.0 (27.0, 39.0) \| 32.0 (25.0, 39.0) \| \| Min, Max \| 12.0, 59.0 \| 13.0, 55.0 \| 12.0, 59.0 \| \| N (% Non-missing) \| 301 (98.4%) \| 303 (99.3%) \| 604 (98.9%) \| \| Endline \|  \|  \|  \| \| Mean (SD) \| 31.0 (9.2) \| 33.2 (9.0) \| 32.1 (9.2) \| \| Median (Q1, Q3) \| 31.0 (24.0, 37.0) \| 34.0 (26.0, 39.0) \| 32.5 (25.0, 39.0) \| \| Min, Max \| 12.0, 52.0 \| 12.0, 57.0 \| 12.0, 57.0 \| \| N (% Non-missing) \| 301 (98.4%) \| 301 (98.7%) \| 602 (98.5%) \| \| **SSS (items 1-8) total (8-40)** \|  \|  \|  \| \| Baseline \|  \|  \|  \| \| Mean (SD) \| 23.0 (6.7) \| 23.8 (6.9) \| 23.4 (6.8) \| \| Median (Q1, Q3) \| 23.0 (18.0, 28.0) \| 24.0 (18.0, 29.0) \| 23.0 (18.0, 29.0) \| \| Min, Max \| 8.0, 40.0 \| 10.0, 40.0 \| 8.0, 40.0 \| \| N (% Non-missing) \| 306 (100.0%) \| 305 (100.0%) \| 611 (100.0%) \| \| Midline \|  \|  \|  \| \| Mean (SD) \| 23.1 (6.9) \| 21.5 (6.9) \| 22.3 (7.0) \| \| Median (Q1, Q3) \| 24.0 (18.0, 28.0) \| 21.0 (16.0, 27.0) \| 22.0 (17.0, 27.0) \| \| Min, Max \| 8.0, 40.0 \| 8.0, 40.0 \| 8.0, 40.0 \| \| N (% Non-missing) \| 301 (98.4%) \| 303 (99.3%) \| 604 (98.9%) \| \| Endline \|  \|  \|  \| \| Mean (SD) \| 22.1 (7.0) \| 21.6 (7.5) \| 21.8 (7.3) \| \| Median (Q1, Q3) \| 22.0 (17.0, 27.0) \| 22.0 (15.0, 27.0) \| 22.0 (16.0, 27.0) \| \| Min, Max \| 8.0, 40.0 \| 8.0, 38.0 \| 8.0, 40.0 \| \| N (% Non-missing) \| 301 (98.4%) \| 301 (98.7%) \| 602 (98.5%) \| \| **RTC (items 1-10) total (10-50)** \|  \|  \|  \| \| Baseline \|  \|  \|  \| \| Mean (SD) \| 26.3 (6.4) \| 27.5 (6.5) \| 26.9 (6.5) \| \| Median (Q1, Q3) \| 26.0 (22.0, 30.0) \| 28.0 (23.0, 32.0) \| 27.0 (22.0, 32.0) \| \| Min, Max \| 10.0, 48.0 \| 10.0, 46.0 \| 10.0, 48.0 \| \| N (% Non-missing) \| 306 (100.0%) \| 305 (100.0%) \| 611 (100.0%) \| \| Midline \|  \|  \|  \| \| Mean (SD) \| 25.9 (6.2) \| 28.5 (6.2) \| 27.2 (6.3) \| \| Median (Q1, Q3) \| 26.0 (21.0, 30.0) \| 29.0 (25.0, 33.0) \| 28.0 (23.0, 32.0) \| \| Min, Max \| 10.0, 45.0 \| 12.0, 44.0 \| 10.0, 45.0 \| \| N (% Non-missing) \| 301 (98.4%) \| 303 (99.3%) \| 604 (98.9%) \| \| Endline \|  \|  \|  \| \| Mean (SD) \| 25.5 (5.5) \| 27.4 (6.3) \| 26.4 (6.0) \| \| Median (Q1, Q3) \| 25.0 (22.0, 29.0) \| 27.0 (24.0, 32.0) \| 26.0 (23.0, 31.0) \| \| Min, Max \| 13.0, 40.0 \| 10.0, 46.0 \| 10.0, 46.0 \| \| N (% Non-missing) \| 301 (98.4%) \| 301 (98.7%) \| 602 (98.5%) \| \| **Have you felt problems in your Heart mind?** \|  \|  \|  \| \| Baseline \|  \|  \|  \| \| Yes \| 306 (100.0%) \| 305 (100.0%) \| 611 (100.0%) \| \| Midline \|  \|  \|  \| \| No \| 72 (23.9%) \| 119 (39.3%) \| 191 (31.6%) \| \| Yes \| 229 (76.1%) \| 184 (60.7%) \| 413 (68.4%) \| \| Endline \|  \|  \|  \| \| No \| 92 (30.6%) \| 124 (41.2%) \| 216 (35.9%) \| \| Yes \| 209 (69.4%) \| 177 (58.8%) \| 386 (64.1%) \| \| **50% reduction in PHQ-9 from baseline to midline** \|  \|  \|  \| \| No \| 255 (84.7%) \| 229 (75.6%) \| 484 (80.1%) \| \| Yes \| 46 (15.3%) \| 74 (24.4%) \| 120 (19.9%) \| \| **50% reduction in PHQ-9 from baseline to endline** \|  \|  \|  \| \| No \| 249 (82.7%) \| 211 (70.1%) \| 460 (76.4%) \| \| Yes \| 52 (17.3%) \| 90 (29.9%) \| 142 (23.6%) \| |

**Table I. Outcome summaries by arm of the completers population**

| \|  \| Control \| PM+ Completer \| PM+ Non-Completer \| Total \| \| --- \| --- \| --- \| --- \| --- \| \|  \| (N = 306) \| (N = 238) \| (N = 67) \| (N = 611) \| |
| --- | --- | --- | --- | --- | --- | --- | --- | --- | --- | --- |
| \| **GHQ-12 total (0-36)** \|  \|  \|  \|  \| \| --- \| --- \| --- \| --- \| --- \| \| Baseline \|  \|  \|  \|  \| \| Mean (SD) \| 20.9 (6.0) \| 21.1 (6.4) \| 21.5 (5.8) \| 21.0 (6.1) \| \| Median (Q1, Q3) \| 21.0 (17.0, 25.0) \| 20.5 (16.0, 26.0) \| 22.0 (17.0, 26.0) \| 21.0 (17.0, 25.0) \| \| Min, Max \| 0.0, 35.0 \| 4.0, 35.0 \| 7.0, 34.0 \| 0.0, 35.0 \| \| N (% Non-missing) \| 306 (100.0%) \| 238 (100.0%) \| 67 (100.0%) \| 611 (100.0%) \| \| Midline \|  \|  \|  \|  \| \| Mean (SD) \| 20.3 (6.6) \| 17.3 (6.7) \| 19.1 (7.4) \| 19.0 (6.9) \| \| Median (Q1, Q3) \| 20.0 (17.0, 25.0) \| 17.0 (13.0, 22.0) \| 19.0 (15.0, 23.0) \| 19.0 (14.0, 23.0) \| \| Min, Max \| 2.0, 36.0 \| 1.0, 35.0 \| 4.0, 34.0 \| 1.0, 36.0 \| \| N (% Non-missing) \| 301 (98.4%) \| 238 (100.0%) \| 65 (97.0%) \| 604 (98.9%) \| \| Endline \|  \|  \|  \|  \| \| Mean (SD) \| 19.3 (6.5) \| 18.1 (6.8) \| 18.1 (7.5) \| 18.7 (6.7) \| \| Median (Q1, Q3) \| 20.0 (15.0, 24.0) \| 18.0 (13.0, 22.0) \| 18.5 (13.0, 23.0) \| 19.0 (14.0, 23.0) \| \| Min, Max \| 1.0, 35.0 \| 2.0, 36.0 \| 0.0, 36.0 \| 0.0, 36.0 \| \| N (% Non-missing) \| 301 (98.4%) \| 237 (99.6%) \| 64 (95.5%) \| 602 (98.5%) \| \| **WHODAS (items 1-12) total (0-48)** \|  \|  \|  \|  \| \| Baseline \|  \|  \|  \|  \| \| Mean (SD) \| 24.6 (6.5) \| 25.9 (6.5) \| 26.2 (7.7) \| 25.3 (6.7) \| \| Median (Q1, Q3) \| 23.0 (19.0, 28.0) \| 25.0 (21.0, 30.0) \| 24.0 (19.0, 32.0) \| 24.0 (20.0, 29.0) \| \| Min, Max \| 17.0, 48.0 \| 17.0, 46.0 \| 17.0, 44.0 \| 17.0, 48.0 \| \| N (% Non-missing) \| 306 (100.0%) \| 238 (100.0%) \| 67 (100.0%) \| 611 (100.0%) \| \| Midline \|  \|  \|  \|  \| \| Mean (SD) \| 19.2 (9.6) \| 16.9 (8.3) \| 17.8 (10.5) \| 18.2 (9.3) \| \| Median (Q1, Q3) \| 19.0 (13.0, 26.0) \| 16.5 (10.0, 24.0) \| 17.0 (11.0, 24.0) \| 18.0 (11.0, 24.0) \| \| Min, Max \| 0.0, 47.0 \| 0.0, 39.0 \| 0.0, 45.0 \| 0.0, 47.0 \| \| N (% Non-missing) \| 301 (98.4%) \| 238 (100.0%) \| 65 (97.0%) \| 604 (98.9%) \| \| Endline \|  \|  \|  \|  \| \| Mean (SD) \| 16.5 (8.6) \| 15.8 (9.5) \| 16.7 (10.1) \| 16.2 (9.1) \| \| Median (Q1, Q3) \| 16.0 (10.0, 22.0) \| 16.0 (8.0, 23.0) \| 17.0 (9.5, 23.0) \| 16.0 (9.0, 23.0) \| \| Min, Max \| 0.0, 45.0 \| 0.0, 45.0 \| 0.0, 41.0 \| 0.0, 45.0 \| \| N (% Non-missing) \| 301 (98.4%) \| 237 (99.6%) \| 64 (95.5%) \| 602 (98.5%) \| \| **PHQ-9 (items 1-10) total (0-30)** \|  \|  \|  \|  \| \| Baseline \|  \|  \|  \|  \| \| Mean (SD) \| 11.9 (5.0) \| 12.7 (4.9) \| 12.6 (4.9) \| 12.3 (5.0) \| \| Median (Q1, Q3) \| 12.0 (8.0, 15.0) \| 12.5 (9.0, 16.0) \| 12.0 (8.0, 16.0) \| 12.0 (9.0, 16.0) \| \| Min, Max \| 0.0, 27.0 \| 1.0, 27.0 \| 4.0, 24.0 \| 0.0, 27.0 \| \| N (% Non-missing) \| 306 (100.0%) \| 238 (100.0%) \| 67 (100.0%) \| 611 (100.0%) \| \| Midline \|  \|  \|  \|  \| \| Mean (SD) \| 10.9 (5.2) \| 9.4 (4.8) \| 10.1 (5.7) \| 10.2 (5.1) \| \| Median (Q1, Q3) \| 11.0 (7.0, 14.0) \| 9.0 (6.0, 13.0) \| 10.0 (6.0, 13.0) \| 10.0 (6.0, 13.0) \| \| Min, Max \| 0.0, 27.0 \| 0.0, 24.0 \| 0.0, 24.0 \| 0.0, 27.0 \| \| N (% Non-missing) \| 301 (98.4%) \| 238 (100.0%) \| 65 (97.0%) \| 604 (98.9%) \| \| Endline \|  \|  \|  \|  \| \| Mean (SD) \| 10.0 (4.4) \| 9.4 (5.2) \| 10.1 (5.3) \| 9.8 (4.8) \| \| Median (Q1, Q3) \| 10.0 (7.0, 13.0) \| 9.0 (5.0, 13.0) \| 10.0 (7.0, 13.0) \| 10.0 (6.0, 13.0) \| \| Min, Max \| 0.0, 26.0 \| 0.0, 24.0 \| 0.0, 25.0 \| 0.0, 26.0 \| \| N (% Non-missing) \| 301 (98.4%) \| 237 (99.6%) \| 64 (95.5%) \| 602 (98.5%) \| \| **PCL (items 1-8) total (8-40)** \|  \|  \|  \|  \| \| Baseline \|  \|  \|  \|  \| \| Mean (SD) \| 21.8 (7.0) \| 23.2 (6.6) \| 22.5 (7.5) \| 22.4 (6.9) \| \| Median (Q1, Q3) \| 21.5 (17.0, 26.0) \| 24.0 (18.0, 28.0) \| 22.0 (16.0, 29.0) \| 22.0 (17.0, 27.0) \| \| Min, Max \| 8.0, 40.0 \| 8.0, 40.0 \| 8.0, 38.0 \| 8.0, 40.0 \| \| N (% Non-missing) \| 306 (100.0%) \| 238 (100.0%) \| 67 (100.0%) \| 611 (100.0%) \| \| Midline \|  \|  \|  \|  \| \| Mean (SD) \| 21.4 (6.9) \| 20.3 (6.6) \| 20.7 (8.0) \| 20.9 (6.9) \| \| Median (Q1, Q3) \| 21.0 (16.0, 26.0) \| 20.0 (15.0, 25.0) \| 20.0 (15.0, 27.0) \| 20.5 (16.0, 26.0) \| \| Min, Max \| 8.0, 40.0 \| 8.0, 37.0 \| 8.0, 39.0 \| 8.0, 40.0 \| \| N (% Non-missing) \| 301 (98.4%) \| 238 (100.0%) \| 65 (97.0%) \| 604 (98.9%) \| \| Endline \|  \|  \|  \|  \| \| Mean (SD) \| 20.5 (6.6) \| 20.3 (7.0) \| 19.9 (7.4) \| 20.3 (6.9) \| \| Median (Q1, Q3) \| 21.0 (16.0, 25.0) \| 20.0 (15.0, 25.0) \| 21.0 (14.0, 25.0) \| 20.0 (15.0, 25.0) \| \| Min, Max \| 8.0, 40.0 \| 8.0, 38.0 \| 8.0, 36.0 \| 8.0, 40.0 \| \| N (% Non-missing) \| 301 (98.4%) \| 237 (99.6%) \| 64 (95.5%) \| 602 (98.5%) \| \| **MSPSS (items 1-12) total (12-60)** \|  \|  \|  \|  \| \| Baseline \|  \|  \|  \|  \| \| Mean (SD) \| 30.7 (9.5) \| 32.3 (9.7) \| 33.5 (10.3) \| 31.6 (9.7) \| \| Median (Q1, Q3) \| 31.0 (23.0, 37.0) \| 32.0 (26.0, 40.0) \| 34.0 (26.0, 41.0) \| 32.0 (24.0, 38.0) \| \| Min, Max \| 12.0, 57.0 \| 12.0, 57.0 \| 12.0, 56.0 \| 12.0, 57.0 \| \| N (% Non-missing) \| 306 (100.0%) \| 238 (100.0%) \| 67 (100.0%) \| 611 (100.0%) \| \| Midline \|  \|  \|  \|  \| \| Mean (SD) \| 30.8 (9.5) \| 32.8 (8.7) \| 33.4 (8.8) \| 31.9 (9.1) \| \| Median (Q1, Q3) \| 31.0 (24.0, 38.0) \| 33.0 (27.0, 39.0) \| 33.0 (28.0, 38.0) \| 32.0 (25.0, 39.0) \| \| Min, Max \| 12.0, 59.0 \| 13.0, 55.0 \| 15.0, 52.0 \| 12.0, 59.0 \| \| N (% Non-missing) \| 301 (98.4%) \| 238 (100.0%) \| 65 (97.0%) \| 604 (98.9%) \| \| Endline \|  \|  \|  \|  \| \| Mean (SD) \| 31.0 (9.2) \| 32.5 (8.8) \| 35.4 (9.5) \| 32.1 (9.2) \| \| Median (Q1, Q3) \| 31.0 (24.0, 37.0) \| 33.0 (26.0, 39.0) \| 34.5 (30.0, 41.0) \| 32.5 (25.0, 39.0) \| \| Min, Max \| 12.0, 52.0 \| 12.0, 53.0 \| 15.0, 57.0 \| 12.0, 57.0 \| \| N (% Non-missing) \| 301 (98.4%) \| 237 (99.6%) \| 64 (95.5%) \| 602 (98.5%) \| \| **SSS (items 1-8) total (8-40)** \|  \|  \|  \|  \| \| Baseline \|  \|  \|  \|  \| \| Mean (SD) \| 23.0 (6.7) \| 24.0 (6.8) \| 22.8 (7.4) \| 23.4 (6.8) \| \| Median (Q1, Q3) \| 23.0 (18.0, 28.0) \| 24.0 (18.0, 29.0) \| 23.0 (16.0, 30.0) \| 23.0 (18.0, 29.0) \| \| Min, Max \| 8.0, 40.0 \| 11.0, 40.0 \| 10.0, 37.0 \| 8.0, 40.0 \| \| N (% Non-missing) \| 306 (100.0%) \| 238 (100.0%) \| 67 (100.0%) \| 611 (100.0%) \| \| Midline \|  \|  \|  \|  \| \| Mean (SD) \| 23.1 (6.9) \| 21.5 (6.6) \| 21.1 (7.8) \| 22.3 (7.0) \| \| Median (Q1, Q3) \| 24.0 (18.0, 28.0) \| 21.0 (17.0, 26.0) \| 22.0 (14.0, 27.0) \| 22.0 (17.0, 27.0) \| \| Min, Max \| 8.0, 40.0 \| 8.0, 40.0 \| 8.0, 36.0 \| 8.0, 40.0 \| \| N (% Non-missing) \| 301 (98.4%) \| 238 (100.0%) \| 65 (97.0%) \| 604 (98.9%) \| \| Endline \|  \|  \|  \|  \| \| Mean (SD) \| 22.1 (7.0) \| 21.7 (7.4) \| 21.0 (8.2) \| 21.8 (7.3) \| \| Median (Q1, Q3) \| 22.0 (17.0, 27.0) \| 22.0 (15.0, 28.0) \| 21.0 (15.0, 26.5) \| 22.0 (16.0, 27.0) \| \| Min, Max \| 8.0, 40.0 \| 8.0, 38.0 \| 8.0, 38.0 \| 8.0, 40.0 \| \| N (% Non-missing) \| 301 (98.4%) \| 237 (99.6%) \| 64 (95.5%) \| 602 (98.5%) \| \| **RTC (items 1-10) total (10-50)** \|  \|  \|  \|  \| \| Baseline \|  \|  \|  \|  \| \| Mean (SD) \| 26.3 (6.4) \| 27.5 (6.6) \| 27.5 (6.0) \| 26.9 (6.5) \| \| Median (Q1, Q3) \| 26.0 (22.0, 30.0) \| 27.0 (23.0, 33.0) \| 28.0 (24.0, 32.0) \| 27.0 (22.0, 32.0) \| \| Min, Max \| 10.0, 48.0 \| 10.0, 46.0 \| 16.0, 42.0 \| 10.0, 48.0 \| \| N (% Non-missing) \| 306 (100.0%) \| 238 (100.0%) \| 67 (100.0%) \| 611 (100.0%) \| \| Midline \|  \|  \|  \|  \| \| Mean (SD) \| 25.9 (6.2) \| 28.5 (6.0) \| 28.6 (6.9) \| 27.2 (6.3) \| \| Median (Q1, Q3) \| 26.0 (21.0, 30.0) \| 29.0 (25.0, 32.0) \| 29.0 (25.0, 34.0) \| 28.0 (23.0, 32.0) \| \| Min, Max \| 10.0, 45.0 \| 12.0, 43.0 \| 14.0, 44.0 \| 10.0, 45.0 \| \| N (% Non-missing) \| 301 (98.4%) \| 238 (100.0%) \| 65 (97.0%) \| 604 (98.9%) \| \| Endline \|  \|  \|  \|  \| \| Mean (SD) \| 25.5 (5.5) \| 27.3 (6.3) \| 28.0 (6.0) \| 26.4 (6.0) \| \| Median (Q1, Q3) \| 25.0 (22.0, 29.0) \| 27.0 (23.0, 32.0) \| 28.5 (24.5, 32.5) \| 26.0 (23.0, 31.0) \| \| Min, Max \| 13.0, 40.0 \| 11.0, 46.0 \| 10.0, 41.0 \| 10.0, 46.0 \| \| N (% Non-missing) \| 301 (98.4%) \| 237 (99.6%) \| 64 (95.5%) \| 602 (98.5%) \| \| **Have you felt problems inyour Heart mind?** \|  \|  \|  \|  \| \| Baseline \|  \|  \|  \|  \| \| Yes \| 306 (100.0%) \| 238 (100.0%) \| 67 (100.0%) \| 611 (100.0%) \| \| Midline \|  \|  \|  \|  \| \| No \| 72 (23.9%) \| 103 (43.3%) \| 16 (24.6%) \| 191 (31.6%) \| \| Yes \| 229 (76.1%) \| 135 (56.7%) \| 49 (75.4%) \| 413 (68.4%) \| \| Endline \|  \|  \|  \|  \| \| No \| 92 (30.6%) \| 95 (40.1%) \| 29 (45.3%) \| 216 (35.9%) \| \| Yes \| 209 (69.4%) \| 142 (59.9%) \| 35 (54.7%) \| 386 (64.1%) \| \| **50% reduction in PHQ-9 from baseline to midline** \|  \|  \|  \|  \| \| No \| 255 (84.7%) \| 178 (74.8%) \| 51 (78.5%) \| 484 (80.1%) \| \| Yes \| 46 (15.3%) \| 60 (25.2%) \| 14 (21.5%) \| 120 (19.9%) \| \| **50% reduction in PHQ-9 from baseline to endline** \|  \|  \|  \|  \| \| No \| 249 (82.7%) \| 165 (69.6%) \| 46 (71.9%) \| 460 (76.4%) \| \| Yes \| 52 (17.3%) \| 72 (30.4%) \| 18 (28.1%) \| 142 (23.6%) \| |

**Table J. Baseline characteristics by arm and by midline data availability**

| \|  \| Control-Missing \| Control-Not Missing \| Intervention-Missing \| Intervention-Not Missing \| Total \| \| --- \| --- \| --- \| --- \| --- \| --- \| \|  \| (N = 5) \| (N = 301) \| (N = 2) \| (N = 303) \| (N = 611) \| |
| --- | --- | --- | --- | --- | --- | --- | --- | --- | --- | --- | --- | --- |
| \| **Age (years)** \|  \|  \|  \|  \|  \| \| --- \| --- \| --- \| --- \| --- \| --- \| \| Mean (SD) \| 41.4 (10.7) \| 44.1 (14.0) \| 47.5 (26.2) \| 45.4 (14.8) \| 44.8 (14.4) \| \| Median (Q1, Q3) \| 45.0 (35.0, 50.0) \| 45.0 (33.0, 54.0) \| 47.5 (29.0, 66.0) \| 44.0 (35.0, 55.0) \| 45.0 (34.0, 55.0) \| \| Min, Max \| 26.0, 51.0 \| 18.0, 83.0 \| 29.0, 66.0 \| 18.0, 91.0 \| 18.0, 91.0 \| \| **Age categories (years)** \|  \|  \|  \|  \|  \| \| < 30 \| 1 (20.0%) \| 49 (16.3%) \| 1 (50.0%) \| 37 (12.2%) \| 88 (14.4%) \| \| 30 - < 40 \| 1 (20.0%) \| 68 (22.6%) \| 0 (0.0%) \| 78 (25.7%) \| 147 (24.1%) \| \| 40 - < 50 \| 1 (20.0%) \| 78 (25.9%) \| 0 (0.0%) \| 72 (23.8%) \| 151 (24.7%) \| \| 50 - < 60 \| 2 (40.0%) \| 60 (19.9%) \| 0 (0.0%) \| 57 (18.8%) \| 119 (19.5%) \| \| 60 - <70 \| 0 (0.0%) \| 34 (11.3%) \| 1 (50.0%) \| 37 (12.2%) \| 72 (11.8%) \| \| 70+ \| 0 (0.0%) \| 12 (4.0%) \| 0 (0.0%) \| 22 (7.3%) \| 34 (5.6%) \| \| **Gender** \|  \|  \|  \|  \|  \| \| Male \| 3 (60.0%) \| 52 (17.3%) \| 1 (50.0%) \| 53 (17.5%) \| 109 (17.8%) \| \| Female \| 2 (40.0%) \| 249 (82.7%) \| 1 (50.0%) \| 250 (82.5%) \| 502 (82.2%) \| \| **Education** \|  \|  \|  \|  \|  \| \| Can’t read or write \| 3 (60.0%) \| 85 (28.2%) \| 1 (50.0%) \| 87 (28.7%) \| 176 (28.8%) \| \| Literate or informal education \| 1 (20.0%) \| 83 (27.6%) \| 0 (0.0%) \| 80 (26.4%) \| 164 (26.8%) \| \| Primary level \| 0 (0.0%) \| 72 (23.9%) \| 0 (0.0%) \| 77 (25.4%) \| 149 (24.4%) \| \| Secondary \| 1 (20.0%) \| 46 (15.3%) \| 1 (50.0%) \| 44 (14.5%) \| 92 (15.1%) \| \| Higher secondary \| 0 (0.0%) \| 13 (4.3%) \| 0 (0.0%) \| 13 (4.3%) \| 26 (4.3%) \| \| University \| 0 (0.0%) \| 2 (0.7%) \| 0 (0.0%) \| 2 (0.7%) \| 4 (0.7%) \| \| **Occupation** \|  \|  \|  \|  \|  \| \| Farmer \| 0 (0.0%) \| 104 (34.6%) \| 1 (50.0%) \| 92 (30.4%) \| 197 (32.2%) \| \| Business or Job \| 1 (20.0%) \| 32 (10.6%) \| 0 (0.0%) \| 34 (11.2%) \| 67 (10.9%) \| \| Daily wage laborer \| 1 (20.0%) \| 34 (11.3%) \| 0 (0.0%) \| 33 (10.9%) \| 68 (11.1%) \| \| Unemployed \| 1 (20.0%) \| 8 (2.7%) \| 0 (0.0%) \| 14 (4.6%) \| 23 (3.8%) \| \| Student \| 0 (0.0%) \| 8 (2.7%) \| 0 (0.0%) \| 4 (1.3%) \| 12 (2.0%) \| \| Housewife \| 1 (20.0%) \| 112 (37.2%) \| 1 (50.0%) \| 119 (39.3%) \| 233 (38.1%) \| \| Other \| 1 (20.0%) \| 3 (1.0%) \| 0 (0.0%) \| 7 (2.3%) \| 11 (1.8%) \| \| **Caste** \|  \|  \|  \|  \|  \| \| Brahmin \| 1 (20.0%) \| 54 (17.9%) \| 0 (0.0%) \| 60 (19.8%) \| 115 (18.8%) \| \| Chetri \| 1 (20.0%) \| 54 (17.9%) \| 1 (50.0%) \| 49 (16.2%) \| 105 (17.2%) \| \| Tharu \| 0 (0.0%) \| 29 (9.6%) \| 0 (0.0%) \| 35 (11.6%) \| 64 (10.5%) \| \| Rai \| 0 (0.0%) \| 29 (9.6%) \| 0 (0.0%) \| 21 (6.9%) \| 50 (8.2%) \| \| Musalman \| 0 (0.0%) \| 1 (0.3%) \| 0 (0.0%) \| 0 (0.0%) \| 1 (0.2%) \| \| Limbu \| 0 (0.0%) \| 22 (7.3%) \| 0 (0.0%) \| 18 (5.9%) \| 40 (6.5%) \| \| Rajbansi \| 0 (0.0%) \| 4 (1.3%) \| 0 (0.0%) \| 1 (0.3%) \| 5 (0.8%) \| \| Newar \| 0 (0.0%) \| 16 (5.3%) \| 0 (0.0%) \| 9 (3.0%) \| 25 (4.1%) \| \| Musahar \| 0 (0.0%) \| 5 (1.7%) \| 0 (0.0%) \| 4 (1.3%) \| 9 (1.5%) \| \| Tamang \| 0 (0.0%) \| 7 (2.3%) \| 0 (0.0%) \| 5 (1.7%) \| 12 (2.0%) \| \| Dhimal \| 0 (0.0%) \| 4 (1.3%) \| 1 (50.0%) \| 5 (1.7%) \| 10 (1.6%) \| \| Magar \| 0 (0.0%) \| 4 (1.3%) \| 0 (0.0%) \| 20 (6.6%) \| 24 (3.9%) \| \| Satar \| 0 (0.0%) \| 0 (0.0%) \| 0 (0.0%) \| 3 (1.0%) \| 3 (0.5%) \| \| Mandal \| 0 (0.0%) \| 2 (0.7%) \| 0 (0.0%) \| 0 (0.0%) \| 2 (0.3%) \| \| Danuwar \| 0 (0.0%) \| 1 (0.3%) \| 0 (0.0%) \| 2 (0.7%) \| 3 (0.5%) \| \| Jhaa \| 0 (0.0%) \| 0 (0.0%) \| 0 (0.0%) \| 1 (0.3%) \| 1 (0.2%) \| \| Jhaangad \| 0 (0.0%) \| 2 (0.7%) \| 0 (0.0%) \| 5 (1.7%) \| 7 (1.1%) \| \| Other \| 3 (60.0%) \| 67 (22.3%) \| 0 (0.0%) \| 65 (21.5%) \| 135 (22.1%) \| \| **Religion** \|  \|  \|  \|  \|  \| \| Hindu \| 5 (100.0%) \| 252 (83.7%) \| 2 (100.0%) \| 265 (87.5%) \| 524 (85.8%) \| \| Buddhist \| 0 (0.0%) \| 10 (3.3%) \| 0 (0.0%) \| 8 (2.6%) \| 18 (2.9%) \| \| Muslim \| 0 (0.0%) \| 1 (0.3%) \| 0 (0.0%) \| 0 (0.0%) \| 1 (0.2%) \| \| Christian \| 0 (0.0%) \| 19 (6.3%) \| 0 (0.0%) \| 17 (5.6%) \| 36 (5.9%) \| \| No religion \| 0 (0.0%) \| 1 (0.3%) \| 0 (0.0%) \| 1 (0.3%) \| 2 (0.3%) \| \| Other \| 0 (0.0%) \| 18 (6.0%) \| 0 (0.0%) \| 12 (4.0%) \| 30 (4.9%) \| \| **Martial status** \|  \|  \|  \|  \|  \| \| Unmarried \| 1 (20.0%) \| 15 (5.0%) \| 0 (0.0%) \| 17 (5.6%) \| 33 (5.4%) \| \| Married \| 4 (80.0%) \| 245 (81.4%) \| 2 (100.0%) \| 240 (79.2%) \| 491 (80.4%) \| \| Widowed \| 0 (0.0%) \| 27 (9.0%) \| 0 (0.0%) \| 39 (12.9%) \| 66 (10.8%) \| \| Divorced \| 0 (0.0%) \| 3 (1.0%) \| 0 (0.0%) \| 3 (1.0%) \| 6 (1.0%) \| \| Separated \| 0 (0.0%) \| 11 (3.7%) \| 0 (0.0%) \| 4 (1.3%) \| 15 (2.5%) \| \| **Most used language** \|  \|  \|  \|  \|  \| \| Nepali \| 5 (100.0%) \| 247 (82.1%) \| 1 (50.0%) \| 248 (81.8%) \| 501 (82.0%) \| \| Maithali \| 0 (0.0%) \| 8 (2.7%) \| 0 (0.0%) \| 3 (1.0%) \| 11 (1.8%) \| \| Tharu \| 0 (0.0%) \| 27 (9.0%) \| 0 (0.0%) \| 36 (11.9%) \| 63 (10.3%) \| \| Rajbansi or Dhimal \| 0 (0.0%) \| 6 (1.9%) \| 1 (50.0%) \| 2 (0.6%) \| 9 (1.4%) \| \| Rai/Limbu,Tamang \| 0 (0.0%) \| 5 (1.9%) \| 0 (0.0%) \| 0 (0.0%) \| 5 (0.8%) \| \| Other \| 0 (0.0%) \| 8 (2.7%) \| 0 (0.0%) \| 14 (4.6%) \| 22 (3.6%) \| \| **Number of members in household** \|  \|  \|  \|  \|  \| \| Living alone \| 0 (0.0%) \| 11 (3.7%) \| 0 (0.0%) \| 9 (3.0%) \| 20 (3.3%) \| \| With 1 other person \| 1 (20.0%) \| 28 (9.3%) \| 0 (0.0%) \| 29 (9.6%) \| 58 (9.5%) \| \| With 2 – 3 other people \| 1 (20.0%) \| 124 (41.2%) \| 1 (50.0%) \| 121 (39.9%) \| 247 (40.4%) \| \| With 4 or more people \| 3 (60.0%) \| 138 (45.8%) \| 1 (50.0%) \| 144 (47.5%) \| 286 (46.8%) \| \| **Chronic diseases** \|  \|  \|  \|  \|  \| \| No \| 3 (60.0%) \| 209 (69.4%) \| 1 (50.0%) \| 208 (68.6%) \| 421 (68.9%) \| \| Yes \| 2 (40.0%) \| 92 (30.6%) \| 1 (50.0%) \| 95 (31.4%) \| 190 (31.1%) \| \| **If yes to chronic disease** \|  \|  \|  \|  \|  \| \| Cancer \| 0 (0.0%) \| 2 (2.2%) \| 0 (0.0%) \| 3 (3.2%) \| 5 (2.6%) \| \| Diabetes \| 1 (50.0%) \| 16 (17.4%) \| 0 (0.0%) \| 18 (18.9%) \| 35 (18.4%) \| \| Hypertension \| 1 (50.0%) \| 35 (38.0%) \| 0 (0.0%) \| 36 (37.9%) \| 72 (37.9%) \| \| Asthma \| 0 (0.0%) \| 14 (15.2%) \| 1 (100.0%) \| 20 (21.1%) \| 35 (18.4%) \| \| Other \| 0 (0.0%) \| 25 (27.2%) \| 0 (0.0%) \| 18 (18.9%) \| 43 (22.6%) \| \| **Who do you live with** \|  \|  \|  \|  \|  \| \| Extended family with spouse \| 3 (60.0%) \| 98 (32.6%) \| 1 (50.0%) \| 80 (26.4%) \| 182 (29.8%) \| \| Extended family without spouse \| 0 (0.0%) \| 15 (5.0%) \| 0 (0.0%) \| 23 (7.6%) \| 38 (6.2%) \| \| With parents \| 0 (0.0%) \| 10 (3.3%) \| 0 (0.0%) \| 9 (3.0%) \| 19 (3.1%) \| \| Maternal home (*maiti*) \| 0 (0.0%) \| 6 (2.0%) \| 0 (0.0%) \| 5 (1.7%) \| 11 (1.8%) \| \| Spouse only \| 0 (0.0%) \| 20 (6.6%) \| 0 (0.0%) \| 17 (5.6%) \| 37 (6.1%) \| \| Spouse and children only \| 1 (20.0%) \| 106 (35.2%) \| 1 (50.0%) \| 115 (38.0%) \| 223 (36.5%) \| \| Other \| 1 (20.0%) \| 46 (15.3%) \| 0 (0.0%) \| 54 (17.8%) \| 101 (16.5%) \| \| **Concrete Building** \|  \|  \|  \|  \|  \| \| No \| 5 (100.0%) \| 262 (87.0%) \| 2 (100.0%) \| 255 (84.2%) \| 524 (85.8%) \| \| Yes \| 0 (0.0%) \| 39 (13.0%) \| 0 (0.0%) \| 48 (15.8%) \| 87 (14.2%) \| \| **Electricity** \|  \|  \|  \|  \|  \| \| No \| 0 (0.0%) \| 35 (11.6%) \| 0 (0.0%) \| 28 (9.2%) \| 63 (10.3%) \| \| Yes \| 5 (100.0%) \| 266 (88.4%) \| 2 (100.0%) \| 275 (90.8%) \| 548 (89.7%) \| \| **Drinking water** \|  \|  \|  \|  \|  \| \| No \| 0 (0.0%) \| 30 (10.0%) \| 0 (0.0%) \| 35 (11.6%) \| 65 (10.6%) \| \| Yes \| 5 (100.0%) \| 271 (90.0%) \| 2 (100.0%) \| 268 (88.4%) \| 546 (89.4%) \| \| **Radio** \|  \|  \|  \|  \|  \| \| No \| 4 (80.0%) \| 217 (72.1%) \| 2 (100.0%) \| 225 (74.3%) \| 448 (73.3%) \| \| Yes \| 1 (20.0%) \| 84 (27.9%) \| 0 (0.0%) \| 78 (25.7%) \| 163 (26.7%) \| \| **Television** \|  \|  \|  \|  \|  \| \| No \| 2 (40.0%) \| 118 (39.2%) \| 2 (100.0%) \| 113 (37.3%) \| 235 (38.5%) \| \| Yes \| 3 (60.0%) \| 183 (60.8%) \| 0 (0.0%) \| 190 (62.7%) \| 376 (61.5%) \| \| **Simple mobile phone** \|  \|  \|  \|  \|  \| \| No \| 2 (40.0%) \| 52 (17.3%) \| 1 (50.0%) \| 62 (20.5%) \| 117 (19.1%) \| \| Yes \| 3 (60.0%) \| 249 (82.7%) \| 1 (50.0%) \| 241 (79.5%) \| 494 (80.9%) \| \| **Smart mobile phone** \|  \|  \|  \|  \|  \| \| No \| 2 (40.0%) \| 153 (50.8%) \| 2 (100.0%) \| 134 (45.1%) \| 291 (48.1%) \| \| Yes \| 3 (60.0%) \| 148 (49.2%) \| 0 (0.0%) \| 163 (54.9%) \| 314 (51.9%) \| \| **Cycle** \|  \|  \|  \|  \|  \| \| No \| 2 (40.0%) \| 102 (33.9%) \| 0 (0.0%) \| 79 (26.1%) \| 183 (30.0%) \| \| Yes \| 3 (60.0%) \| 199 (66.1%) \| 2 (100.0%) \| 224 (73.9%) \| 428 (70.0%) \| \| **LP Gas** \|  \|  \|  \|  \|  \| \| No \| 3 (60.0%) \| 79 (26.2%) \| 0 (0.0%) \| 74 (24.4%) \| 156 (25.5%) \| \| Yes \| 2 (40.0%) \| 222 (73.8%) \| 2 (100.0%) \| 229 (75.6%) \| 455 (74.5%) \| |

**Table K. Baseline characteristics by arm and by endline data availability**

| \|  \| Control-Missing \| Control-Not Missing \| Intervention-Missing \| Intervention-Not Missing \| Total \| \| --- \| --- \| --- \| --- \| --- \| --- \| \|  \| (N = 5) \| (N = 301) \| (N = 4) \| (N = 301) \| (N = 611) \| |
| --- | --- | --- | --- | --- | --- | --- | --- | --- | --- | --- | --- | --- |
| \| **Age (years)** \|  \|  \|  \|  \|  \| \| --- \| --- \| --- \| --- \| --- \| --- \| \| Mean (SD) \| 41.6 (10.5) \| 44.1 (14.0) \| 52.2 (17.1) \| 45.4 (14.8) \| 44.8 (14.4) \| \| Median (Q1, Q3) \| 45.0 (36.0, 50.0) \| 45.0 (33.0, 54.0) \| 57.0 (39.5, 65.0) \| 44.0 (35.0, 55.0) \| 45.0 (34.0, 55.0) \| \| Min, Max \| 26.0, 51.0 \| 18.0, 83.0 \| 29.0, 66.0 \| 18.0, 91.0 \| 18.0, 91.0 \| \| **Age categories (years)** \|  \|  \|  \|  \|  \| \| < 30 \| 1 (20.0%) \| 49 (16.3%) \| 1 (25.0%) \| 37 (12.3%) \| 88 (14.4%) \| \| 30 - < 40 \| 1 (20.0%) \| 68 (22.6%) \| 0 (0.0%) \| 78 (25.9%) \| 147 (24.1%) \| \| 40 - < 50 \| 1 (20.0%) \| 78 (25.9%) \| 0 (0.0%) \| 72 (23.9%) \| 151 (24.7%) \| \| 50 - < 60 \| 2 (40.0%) \| 60 (19.9%) \| 1 (25.0%) \| 56 (18.6%) \| 119 (19.5%) \| \| 60 - <70 \| 0 (0.0%) \| 34 (11.3%) \| 2 (50.0%) \| 36 (12.0%) \| 72 (11.8%) \| \| 70+ \| 0 (0.0%) \| 12 (4.0%) \| 0 (0.0%) \| 22 (7.3%) \| 34 (5.6%) \| \| **Gender** \|  \|  \|  \|  \|  \| \| Male \| 2 (40.0%) \| 53 (17.6%) \| 2 (50.0%) \| 52 (17.3%) \| 109 (17.8%) \| \| Female \| 3 (60.0%) \| 248 (82.4%) \| 2 (50.0%) \| 249 (82.7%) \| 502 (82.2%) \| \| **Education** \|  \|  \|  \|  \|  \| \| Cant read or write \| 3 (60.0%) \| 85 (28.2%) \| 2 (50.0%) \| 86 (28.6%) \| 176 (28.8%) \| \| Literate or informal education \| 1 (20.0%) \| 83 (27.6%) \| 1 (25.0%) \| 79 (26.2%) \| 164 (26.8%) \| \| Primary level \| 0 (0.0%) \| 72 (23.9%) \| 0 (0.0%) \| 77 (25.6%) \| 149 (24.4%) \| \| Secondary \| 0 (0.0%) \| 47 (15.6%) \| 1 (25.0%) \| 44 (14.6%) \| 92 (15.1%) \| \| Higher secondary \| 1 (20.0%) \| 12 (4.0%) \| 0 (0.0%) \| 13 (4.3%) \| 26 (4.3%) \| \| University \| 0 (0.0%) \| 2 (0.7%) \| 0 (0.0%) \| 2 (0.7%) \| 4 (0.7%) \| \| **Occupation** \|  \|  \|  \|  \|  \| \| Farmer \| 0 (0.0%) \| 104 (34.6%) \| 1 (25.0%) \| 92 (30.6%) \| 197 (32.2%) \| \| Business or Job \| 1 (20.0%) \| 32 (9.3%) \| 0 (0.0%) \| 34 (9.3%) \| 67 (9.3%) \| \| Daily wage laborer \| 1 (20.0%) \| 34 (11.3%) \| 1 (25.0%) \| 32 (10.6%) \| 68 (11.1%) \| \| Unemployed \| 0 (0.0%) \| 9 (3.0%) \| 0 (0.0%) \| 14 (4.7%) \| 23 (3.8%) \| \| Student \| 0 (0.0%) \| 8 (2.7%) \| 0 (0.0%) \| 4 (1.3%) \| 12 (2.0%) \| \| Housewife \| 2 (40.0%) \| 111 (36.9%) \| 1 (25.0%) \| 119 (39.5%) \| 233 (38.1%) \| \| Other \| 1 (20.0%) \| 3 (1.0%) \| 1 (25.0%) \| 6 (2.0%) \| 11 (1.8%) \| \| **Caste** \|  \|  \|  \|  \|  \| \| Brahmin \| 1 (20.0%) \| 54 (17.9%) \| 0 (0.0%) \| 60 (19.9%) \| 115 (18.8%) \| \| Chetri \| 1 (20.0%) \| 54 (17.9%) \| 3 (75.0%) \| 47 (15.6%) \| 105 (17.2%) \| \| Tharu \| 0 (0.0%) \| 29 (9.6%) \| 0 (0.0%) \| 35 (11.6%) \| 64 (10.5%) \| \| Rai \| 0 (0.0%) \| 29 (9.6%) \| 0 (0.0%) \| 21 (7.0%) \| 50 (8.2%) \| \| Musalman \| 0 (0.0%) \| 1 (0.3%) \| 0 (0.0%) \| 0 (0.0%) \| 1 (0.2%) \| \| Limbu \| 0 (0.0%) \| 22 (7.3%) \| 0 (0.0%) \| 18 (6.0%) \| 40 (6.5%) \| \| Rajbansi \| 0 (0.0%) \| 4 (1.3%) \| 0 (0.0%) \| 1 (0.3%) \| 5 (0.8%) \| \| Newar \| 0 (0.0%) \| 16 (5.3%) \| 0 (0.0%) \| 9 (3.0%) \| 25 (4.1%) \| \| Musahar \| 0 (0.0%) \| 5 (1.7%) \| 0 (0.0%) \| 4 (1.3%) \| 9 (1.5%) \| \| Tamang \| 0 (0.0%) \| 7 (2.3%) \| 0 (0.0%) \| 5 (1.7%) \| 12 (2.0%) \| \| Dhimal \| 0 (0.0%) \| 4 (1.3%) \| 1 (25.0%) \| 5 (1.7%) \| 10 (1.6%) \| \| Magar \| 0 (0.0%) \| 4 (1.3%) \| 0 (0.0%) \| 20 (6.6%) \| 24 (3.9%) \| \| Satar \| 0 (0.0%) \| 0 (0.0%) \| 0 (0.0%) \| 3 (1.0%) \| 3 (0.5%) \| \| Mandal \| 0 (0.0%) \| 2 (0.7%) \| 0 (0.0%) \| 0 (0.0%) \| 2 (0.3%) \| \| Danuwar \| 0 (0.0%) \| 1 (0.3%) \| 0 (0.0%) \| 2 (0.7%) \| 3 (0.5%) \| \| Jhaa \| 0 (0.0%) \| 0 (0.0%) \| 0 (0.0%) \| 1 (0.3%) \| 1 (0.2%) \| \| Jhaangad \| 0 (0.0%) \| 2 (0.7%) \| 0 (0.0%) \| 5 (1.7%) \| 7 (1.1%) \| \| Other \| 3 (60.0%) \| 67 (22.3%) \| 0 (0.0%) \| 65 (21.6%) \| 135 (22.1%) \| \| **Caste categories** \|  \|  \|  \|  \|  \| \| Brahmin \| 1 (20.0%) \| 54 (17.9%) \| 0 (0.0%) \| 60 (19.9%) \| 115 (18.8%) \| \| Chetri \| 1 (20.0%) \| 54 (17.9%) \| 3 (75.0%) \| 47 (15.6%) \| 105 (17.2%) \| \| Tharu \| 0 (0.0%) \| 29 (9.6%) \| 0 (0.0%) \| 35 (11.6%) \| 64 (10.5%) \| \| Rai \| 0 (0.0%) \| 29 (9.6%) \| 0 (0.0%) \| 21 (7.0%) \| 50 (8.2%) \| \| More \| 0 (0.0%) \| 68 (22.6%) \| 1 (25.0%) \| 73 (24.3%) \| 142 (23.2%) \| \| Other \| 3 (60.0%) \| 67 (22.3%) \| 0 (0.0%) \| 65 (21.6%) \| 135 (22.1%) \| \| **Religion** \|  \|  \|  \|  \|  \| \| Hindu \| 5 (100.0%) \| 252 (83.7%) \| 4 (100.0%) \| 263 (87.4%) \| 524 (85.8%) \| \| Buddhist \| 0 (0.0%) \| 10 (3.3%) \| 0 (0.0%) \| 8 (2.7%) \| 18 (2.9%) \| \| Muslim \| 0 (0.0%) \| 1 (0.3%) \| 0 (0.0%) \| 0 (0.0%) \| 1 (0.2%) \| \| Christian \| 0 (0.0%) \| 19 (6.3%) \| 0 (0.0%) \| 17 (5.6%) \| 36 (5.9%) \| \| No religion \| 0 (0.0%) \| 1 (0.3%) \| 0 (0.0%) \| 1 (0.3%) \| 2 (0.3%) \| \| Other \| 0 (0.0%) \| 18 (6.0%) \| 0 (0.0%) \| 12 (4.0%) \| 30 (4.9%) \| \| **Martial status** \|  \|  \|  \|  \|  \| \| Unmarried \| 0 (0.0%) \| 16 (5.3%) \| 0 (0.0%) \| 17 (5.6%) \| 33 (5.4%) \| \| Married \| 5 (100.0%) \| 244 (81.1%) \| 3 (75.0%) \| 239 (79.4%) \| 491 (80.4%) \| \| Widowed \| 0 (0.0%) \| 27 (9.0%) \| 1 (25.0%) \| 38 (12.6%) \| 66 (10.8%) \| \| Divorced \| 0 (0.0%) \| 3 (1.0%) \| 0 (0.0%) \| 3 (1.0%) \| 6 (1.0%) \| \| Separated \| 0 (0.0%) \| 11 (3.7%) \| 0 (0.0%) \| 4 (1.3%) \| 15 (2.5%) \| \| **Most used language** \|  \|  \|  \|  \|  \| \| Nepali \| 5 (100.0%) \| 247 (82.1%) \| 3 (75.0%) \| 246 (81.7%) \| 501 (82.0%) \| \| Maithali \| 0 (0.0%) \| 8 (2.7%) \| 0 (0.0%) \| 3 (1.0%) \| 11 (1.8%) \| \| Tharu \| 0 (0.0%) \| 27 (9.0%) \| 0 (0.0%) \| 36 (12.0%) \| 63 (10.3%) \| \| Rajbansi or Dhimal \| 0 (0.0%) \| 6 (1.9%) \| 1 (25.0%) \| 2 (0.66%) \| 9 (1.4%) \| \| Rai/Limbu or Tamang \| 0 (0.0%) \| 5 (1.6%) \| 0 (0.0%) \| 0 (0.0%) \| 5 (0.8%) \| \| Other \| 0 (0.0%) \| 8 (2.7%) \| 0 (0.0%) \| 14 (4.7%) \| 22 (3.6%) \| \| **Number of members in household** \|  \|  \|  \|  \|  \| \| Living alone \| 0 (0.0%) \| 11 (3.7%) \| 0 (0.0%) \| 9 (3.0%) \| 20 (3.3%) \| \| With 1 other person \| 0 (0.0%) \| 29 (9.6%) \| 0 (0.0%) \| 29 (9.6%) \| 58 (9.5%) \| \| With 2 – 3 people \| 1 (20.0%) \| 124 (41.1%) \| 3 (75.0%) \| 119 (39.5%) \| 247 (40.4%) \| \| With 4 or more people \| 4 (80.0%) \| 134 (44.5%) \| 1 (25.0%) \| 144 (47.8%) \| 286 (46.8%) \| \| **Chronic diseases** \|  \|  \|  \|  \|  \| \| No \| 3 (60.0%) \| 209 (69.4%) \| 2 (50.0%) \| 207 (68.8%) \| 421 (68.9%) \| \| Yes \| 2 (40.0%) \| 92 (30.6%) \| 2 (50.0%) \| 94 (31.2%) \| 190 (31.1%) \| \| **If yes to chronic disease** \|  \|  \|  \|  \|  \| \| Cancer \| 0 (0.0%) \| 2 (2.2%) \| 0 (0.0%) \| 3 (3.2%) \| 5 (2.6%) \| \| Diabetes \| 1 (50.0%) \| 16 (17.4%) \| 0 (0.0%) \| 18 (19.1%) \| 35 (18.4%) \| \| Hypertension \| 1 (50.0%) \| 35 (38.0%) \| 0 (0.0%) \| 36 (38.3%) \| 72 (37.9%) \| \| Asthma \| 0 (0.0%) \| 14 (15.2%) \| 1 (50.0%) \| 20 (21.3%) \| 35 (18.4%) \| \| Other \| 0 (0.0%) \| 25 (27.2%) \| 1 (50.0%) \| 17 (18.1%) \| 43 (22.6%) \| \| **Who do you live with** \|  \|  \|  \|  \|  \| \| Extended family with spouse \| 4 (80.0%) \| 97 (32.2%) \| 1 (25.0%) \| 80 (26.6%) \| 182 (29.8%) \| \| Extended family without spouse \| 0 (0.0%) \| 15 (5.0%) \| 0 (0.0%) \| 23 (7.6%) \| 38 (6.2%) \| \| With parents \| 0 (0.0%) \| 10 (3.3%) \| 0 (0.0%) \| 9 (3.0%) \| 19 (3.1%) \| \| Maternal home (*maiti*) \| 0 (0.0%) \| 6 (2.0%) \| 0 (0.0%) \| 5 (1.7%) \| 11 (1.8%) \| \| Spouse only \| 0 (0.0%) \| 20 (6.6%) \| 0 (0.0%) \| 17 (5.6%) \| 37 (6.1%) \| \| Spouse and children only \| 1 (20.0%) \| 106 (35.2%) \| 2 (50.0%) \| 114 (37.9%) \| 223 (36.5%) \| \| Other \| 0 (0.0%) \| 47 (15.6%) \| 1 (25.0%) \| 53 (17.6%) \| 101 (16.5%) \| \| **Concrete Building** \|  \|  \|  \|  \|  \| \| No \| 4 (80.0%) \| 263 (87.4%) \| 4 (100.0%) \| 253 (84.1%) \| 524 (85.8%) \| \| Yes \| 1 (20.0%) \| 38 (12.6%) \| 0 (0.0%) \| 48 (15.9%) \| 87 (14.2%) \| \| **Electricity** \|  \|  \|  \|  \|  \| \| No \| 0 (0.0%) \| 35 (11.6%) \| 0 (0.0%) \| 28 (9.3%) \| 63 (10.3%) \| \| Yes \| 5 (100.0%) \| 266 (88.4%) \| 4 (100.0%) \| 273 (90.7%) \| 548 (89.7%) \| \| **Drinking water** \|  \|  \|  \|  \|  \| \| No \| 0 (0.0%) \| 30 (10.0%) \| 0 (0.0%) \| 35 (11.6%) \| 65 (10.6%) \| \| Yes \| 5 (100.0%) \| 271 (90.0%) \| 4 (100.0%) \| 266 (88.4%) \| 546 (89.4%) \| \| **Radio** \|  \|  \|  \|  \|  \| \| No \| 4 (80.0%) \| 217 (72.1%) \| 2 (50.0%) \| 225 (74.8%) \| 448 (73.3%) \| \| Yes \| 1 (20.0%) \| 84 (27.9%) \| 2 (50.0%) \| 76 (25.2%) \| 163 (26.7%) \| \| **Television** \|  \|  \|  \|  \|  \| \| No \| 1 (20.0%) \| 119 (39.5%) \| 3 (75.0%) \| 112 (37.2%) \| 235 (38.5%) \| \| Yes \| 4 (80.0%) \| 182 (60.5%) \| 1 (25.0%) \| 189 (62.8%) \| 376 (61.5%) \| \| **Simple mobile phone** \|  \|  \|  \|  \|  \| \| No \| 2 (40.0%) \| 52 (17.3%) \| 2 (50.0%) \| 61 (20.3%) \| 117 (19.1%) \| \| Yes \| 3 (60.0%) \| 249 (82.7%) \| 2 (50.0%) \| 240 (79.7%) \| 494 (80.9%) \| \| **Smart mobile phone** \|  \|  \|  \|  \|  \| \| No \| 2 (40.0%) \| 153 (50.8%) \| 3 (75.0%) \| 133 (45.1%) \| 291 (48.1%) \| \| Yes \| 3 (60.0%) \| 148 (49.2%) \| 1 (25.0%) \| 162 (54.9%) \| 314 (51.9%) \| \| **Cycle** \|  \|  \|  \|  \|  \| \| No \| 2 (40.0%) \| 102 (33.9%) \| 1 (25.0%) \| 78 (25.9%) \| 183 (30.0%) \| \| Yes \| 3 (60.0%) \| 199 (66.1%) \| 3 (75.0%) \| 223 (74.1%) \| 428 (70.0%) \| \| **LP Gas** \|  \|  \|  \|  \|  \| \| No \| 3 (60.0%) \| 79 (26.2%) \| 0 (0.0%) \| 74 (24.6%) \| 156 (25.5%) \| \| Yes \| 2 (40.0%) \| 222 (73.8%) \| 4 (100.0%) \| 227 (75.4%) \| 455 (74.5%) \| |

**Table L. Outcome summaries by gender and arm (i.e. of the ITT population)**

| \|  \| Female in Control \| Female in PM+ \| Male in Control \| Male in PM+ \| Total \| \| --- \| --- \| --- \| --- \| --- \| --- \| \|  \| (N = 251) \| (N = 251) \| (N = 55) \| (N = 54) \| (N = 611) \| |
| --- | --- | --- | --- | --- | --- | --- | --- | --- | --- | --- | --- | --- |
| \| **GHQ-12 total (0-36)** \|  \|  \|  \|  \|  \| \| --- \| --- \| --- \| --- \| --- \| --- \| \| Baseline \|  \|  \|  \|  \|  \| \| Mean (SD) \| 20.9 (5.9) \| 20.8 (6.0) \| 20.9 (6.4) \| 23.3 (7.2) \| 21.0 (6.1) \| \| Median (Q1, Q3) \| 21.0 (17.0, 25.0) \| 20.0 (16.0, 25.0) \| 20.0 (16.0, 26.0) \| 24.5 (20.0, 27.0) \| 21.0 (17.0, 25.0) \| \| Min, Max \| 0.0, 35.0 \| 4.0, 35.0 \| 6.0, 35.0 \| 5.0, 35.0 \| 0.0, 35.0 \| \| N (% Non-missing) \| 251 (100.0%) \| 251 (100.0%) \| 55 (100.0%) \| 54 (100.0%) \| 611 (100.0%) \| \| Midline \|  \|  \|  \|  \|  \| \| Mean (SD) \| 20.6 (6.5) \| 17.3 (6.8) \| 19.1 (7.1) \| 19.5 (7.3) \| 19.0 (6.9) \| \| Median (Q1, Q3) \| 20.0 (17.0, 25.0) \| 17.0 (13.0, 22.0) \| 20.0 (16.5, 23.5) \| 20.0 (15.0, 25.0) \| 19.0 (14.0, 23.0) \| \| Min, Max \| 3.0, 36.0 \| 1.0, 35.0 \| 2.0, 34.0 \| 5.0, 34.0 \| 1.0, 36.0 \| \| N (% Non-missing) \| 249 (99.2%) \| 250 (99.6%) \| 52 (94.5%) \| 53 (98.1%) \| 604 (98.9%) \| \| Endline \|  \|  \|  \|  \|  \| \| Mean (SD) \| 19.6 (6.5) \| 18.0 (6.5) \| 18.1 (6.3) \| 18.7 (8.7) \| 18.7 (6.7) \| \| Median (Q1, Q3) \| 20.0 (15.0, 24.0) \| 18.0 (13.0, 22.0) \| 18.0 (14.0, 22.0) \| 18.5 (12.5, 25.0) \| 19.0 (14.0, 23.0) \| \| Min, Max \| 1.0, 35.0 \| 3.0, 36.0 \| 5.0, 31.0 \| 0.0, 34.0 \| 0.0, 36.0 \| \| N (% Non-missing) \| 248 (98.8%) \| 249 (99.2%) \| 53 (96.4%) \| 52 (96.3%) \| 602 (98.5%) \| \| **WHODAS (items 1-12) total (0-48)** \|  \|  \|  \|  \|  \| \| Baseline \|  \|  \|  \|  \|  \| \| Mean (SD) \| 24.4 (6.2) \| 25.6 (6.4) \| 25.5 (7.9) \| 27.7 (8.3) \| 25.3 (6.7) \| \| Median (Q1, Q3) \| 23.0 (20.0, 27.0) \| 25.0 (20.0, 30.0) \| 24.0 (19.0, 31.0) \| 26.0 (21.0, 32.0) \| 24.0 (20.0, 29.0) \| \| Min, Max \| 17.0, 48.0 \| 17.0, 45.0 \| 17.0, 48.0 \| 17.0, 46.0 \| 17.0, 48.0 \| \| N (% Non-missing) \| 251 (100.0%) \| 251 (100.0%) \| 55 (100.0%) \| 54 (100.0%) \| 611 (100.0%) \| \| Midline \|  \|  \|  \|  \|  \| \| Mean (SD) \| 19.7 (9.5) \| 17.0 (8.6) \| 17.3 (9.9) \| 17.7 (9.9) \| 18.2 (9.3) \| \| Median (Q1, Q3) \| 20.0 (13.0, 26.0) \| 17.0 (10.0, 24.0) \| 16.0 (10.0, 24.0) \| 19.0 (10.0, 24.0) \| 18.0 (11.0, 24.0) \| \| Min, Max \| 0.0, 47.0 \| 0.0, 45.0 \| 0.0, 44.0 \| 0.0, 39.0 \| 0.0, 47.0 \| \| N (% Non-missing) \| 249 (99.2%) \| 250 (99.6%) \| 52 (94.5%) \| 53 (98.1%) \| 604 (98.9%) \| \| Endline \|  \|  \|  \|  \|  \| \| Mean (SD) \| 16.9 (8.7) \| 16.0 (9.4) \| 14.2 (8.0) \| 15.7 (10.6) \| 16.2 (9.1) \| \| Median (Q1, Q3) \| 17.0 (11.0, 23.0) \| 16.0 (9.0, 22.0) \| 13.0 (9.0, 18.0) \| 12.0 (6.5, 25.0) \| 16.0 (9.0, 23.0) \| \| Min, Max \| 0.0, 45.0 \| 0.0, 45.0 \| 0.0, 39.0 \| 0.0, 37.0 \| 0.0, 45.0 \| \| N (% Non-missing) \| 248 (98.8%) \| 249 (99.2%) \| 53 (96.4%) \| 52 (96.3%) \| 602 (98.5%) \| \| **PHQ-9 (items 1-10) total (0-30)** \|  \|  \|  \|  \|  \| \| Baseline \|  \|  \|  \|  \|  \| \| Mean (SD) \| 11.9 (5.0) \| 12.6 (4.7) \| 12.0 (5.4) \| 13.3 (5.7) \| 12.3 (5.0) \| \| Median (Q1, Q3) \| 11.0 (8.0, 15.0) \| 12.0 (9.0, 16.0) \| 12.0 (8.0, 16.0) \| 13.5 (9.0, 17.0) \| 12.0 (9.0, 16.0) \| \| Min, Max \| 0.0, 27.0 \| 1.0, 27.0 \| 0.0, 24.0 \| 1.0, 26.0 \| 0.0, 27.0 \| \| N (% Non-missing) \| 251 (100.0%) \| 251 (100.0%) \| 55 (100.0%) \| 54 (100.0%) \| 611 (100.0%) \| \| Midline \|  \|  \|  \|  \|  \| \| Mean (SD) \| 11.0 (5.1) \| 9.3 (4.9) \| 10.3 (5.5) \| 10.7 (5.3) \| 10.2 (5.1) \| \| Median (Q1, Q3) \| 11.0 (7.0, 14.0) \| 9.0 (6.0, 13.0) \| 10.0 (6.5, 13.5) \| 11.0 (7.0, 14.0) \| 10.0 (6.0, 13.0) \| \| Min, Max \| 0.0, 27.0 \| 0.0, 24.0 \| 0.0, 23.0 \| 0.0, 20.0 \| 0.0, 27.0 \| \| N (% Non-missing) \| 249 (99.2%) \| 250 (99.6%) \| 52 (94.5%) \| 53 (98.1%) \| 604 (98.9%) \| \| Endline \|  \|  \|  \|  \|  \| \| Mean (SD) \| 10.1 (4.4) \| 9.5 (5.2) \| 9.5 (4.3) \| 9.5 (5.4) \| 9.8 (4.8) \| \| Median (Q1, Q3) \| 10.0 (7.0, 13.0) \| 9.0 (6.0, 13.0) \| 9.0 (6.0, 12.0) \| 9.5 (5.0, 13.5) \| 10.0 (6.0, 13.0) \| \| Min, Max \| 0.0, 26.0 \| 0.0, 25.0 \| 1.0, 22.0 \| 0.0, 20.0 \| 0.0, 26.0 \| \| N (% Non-missing) \| 248 (98.8%) \| 249 (99.2%) \| 53 (96.4%) \| 52 (96.3%) \| 602 (98.5%) \| \| **PCL (items 1-8) total (8-40)** \|  \|  \|  \|  \|  \| \| Baseline \|  \|  \|  \|  \|  \| \| Mean (SD) \| 22.2 (6.7) \| 23.2 (6.6) \| 19.7 (7.7) \| 22.5 (7.7) \| 22.4 (6.9) \| \| Median (Q1, Q3) \| 22.0 (18.0, 27.0) \| 23.0 (18.0, 28.0) \| 20.0 (13.0, 24.0) \| 23.0 (16.0, 28.0) \| 22.0 (17.0, 27.0) \| \| Min, Max \| 8.0, 40.0 \| 8.0, 40.0 \| 8.0, 40.0 \| 8.0, 36.0 \| 8.0, 40.0 \| \| N (% Non-missing) \| 251 (100.0%) \| 251 (100.0%) \| 55 (100.0%) \| 54 (100.0%) \| 611 (100.0%) \| \| Midline \|  \|  \|  \|  \|  \| \| Mean (SD) \| 21.9 (6.9) \| 20.1 (6.7) \| 18.8 (6.7) \| 21.9 (7.5) \| 20.9 (6.9) \| \| Median (Q1, Q3) \| 22.0 (17.0, 27.0) \| 20.0 (15.0, 24.0) \| 18.0 (13.5, 23.0) \| 21.0 (16.0, 28.0) \| 20.5 (16.0, 26.0) \| \| Min, Max \| 8.0, 40.0 \| 8.0, 39.0 \| 8.0, 36.0 \| 8.0, 35.0 \| 8.0, 40.0 \| \| N (% Non-missing) \| 249 (99.2%) \| 250 (99.6%) \| 52 (94.5%) \| 53 (98.1%) \| 604 (98.9%) \| \| Endline \|  \|  \|  \|  \|  \| \| Mean (SD) \| 21.0 (6.5) \| 20.3 (7.0) \| 18.0 (6.5) \| 19.8 (7.5) \| 20.3 (6.9) \| \| Median (Q1, Q3) \| 21.5 (16.5, 26.0) \| 20.0 (15.0, 25.0) \| 19.0 (12.0, 22.0) \| 18.5 (14.5, 25.0) \| 20.0 (15.0, 25.0) \| \| Min, Max \| 8.0, 40.0 \| 8.0, 38.0 \| 8.0, 36.0 \| 8.0, 36.0 \| 8.0, 40.0 \| \| N (% Non-missing) \| 248 (98.8%) \| 249 (99.2%) \| 53 (96.4%) \| 52 (96.3%) \| 602 (98.5%) \| \| **MSPSS (items 1-12) total (12-60)** \|  \|  \|  \|  \|  \| \| Baseline \|  \|  \|  \|  \|  \| \| Mean (SD) \| 30.4 (9.7) \| 32.2 (9.8) \| 32.2 (8.4) \| 34.0 (10.1) \| 31.6 (9.7) \| \| Median (Q1, Q3) \| 31.0 (23.0, 37.0) \| 32.0 (26.0, 40.0) \| 32.0 (27.0, 37.0) \| 32.5 (28.0, 41.0) \| 32.0 (24.0, 38.0) \| \| Min, Max \| 12.0, 55.0 \| 12.0, 57.0 \| 17.0, 57.0 \| 16.0, 56.0 \| 12.0, 57.0 \| \| N (% Non-missing) \| 251 (100.0%) \| 251 (100.0%) \| 55 (100.0%) \| 54 (100.0%) \| 611 (100.0%) \| \| Midline \|  \|  \|  \|  \|  \| \| Mean (SD) \| 30.2 (9.6) \| 32.6 (8.5) \| 33.5 (8.3) \| 34.5 (9.4) \| 31.9 (9.1) \| \| Median (Q1, Q3) \| 30.0 (23.0, 38.0) \| 33.0 (27.0, 39.0) \| 34.0 (27.5, 39.5) \| 33.0 (29.0, 41.0) \| 32.0 (25.0, 39.0) \| \| Min, Max \| 12.0, 58.0 \| 13.0, 54.0 \| 14.0, 59.0 \| 13.0, 55.0 \| 12.0, 59.0 \| \| N (% Non-missing) \| 249 (99.2%) \| 250 (99.6%) \| 52 (94.5%) \| 53 (98.1%) \| 604 (98.9%) \| \| Endline \|  \|  \|  \|  \|  \| \| Mean (SD) \| 30.9 (9.2) \| 32.8 (8.6) \| 31.4 (8.9) \| 35.0 (10.7) \| 32.1 (9.2) \| \| Median (Q1, Q3) \| 31.0 (24.0, 37.0) \| 33.0 (27.0, 39.0) \| 30.0 (25.0, 38.0) \| 35.5 (25.0, 44.0) \| 32.5 (25.0, 39.0) \| \| Min, Max \| 12.0, 52.0 \| 12.0, 57.0 \| 12.0, 51.0 \| 16.0, 55.0 \| 12.0, 57.0 \| \| N (% Non-missing) \| 248 (98.8%) \| 249 (99.2%) \| 53 (96.4%) \| 52 (96.3%) \| 602 (98.5%) \| \| **SSS (items 1-8) total (8-40)** \|  \|  \|  \|  \|  \| \| Baseline \|  \|  \|  \|  \|  \| \| Mean (SD) \| 23.5 (6.8) \| 24.3 (6.8) \| 20.7 (5.9) \| 21.4 (7.1) \| 23.4 (6.8) \| \| Median (Q1, Q3) \| 23.0 (19.0, 29.0) \| 24.0 (18.0, 30.0) \| 21.0 (17.0, 25.0) \| 21.0 (16.0, 26.0) \| 23.0 (18.0, 29.0) \| \| Min, Max \| 8.0, 40.0 \| 11.0, 40.0 \| 10.0, 34.0 \| 10.0, 38.0 \| 8.0, 40.0 \| \| N (% Non-missing) \| 251 (100.0%) \| 251 (100.0%) \| 55 (100.0%) \| 54 (100.0%) \| 611 (100.0%) \| \| Midline \|  \|  \|  \|  \|  \| \| Mean (SD) \| 23.8 (6.8) \| 21.8 (6.9) \| 19.8 (6.6) \| 20.0 (6.8) \| 22.3 (7.0) \| \| Median (Q1, Q3) \| 24.0 (19.0, 28.0) \| 21.5 (17.0, 27.0) \| 19.0 (14.0, 25.0) \| 19.0 (14.0, 26.0) \| 22.0 (17.0, 27.0) \| \| Min, Max \| 9.0, 40.0 \| 8.0, 40.0 \| 8.0, 33.0 \| 8.0, 32.0 \| 8.0, 40.0 \| \| N (% Non-missing) \| 249 (99.2%) \| 250 (99.6%) \| 52 (94.5%) \| 53 (98.1%) \| 604 (98.9%) \| \| Endline \|  \|  \|  \|  \|  \| \| Mean (SD) \| 22.8 (7.0) \| 22.2 (7.5) \| 18.9 (6.0) \| 18.7 (7.1) \| 21.8 (7.3) \| \| Median (Q1, Q3) \| 23.0 (17.0, 28.0) \| 23.0 (16.0, 28.0) \| 19.0 (14.0, 23.0) \| 18.0 (13.0, 23.5) \| 22.0 (16.0, 27.0) \| \| Min, Max \| 8.0, 40.0 \| 8.0, 38.0 \| 8.0, 34.0 \| 8.0, 34.0 \| 8.0, 40.0 \| \| N (% Non-missing) \| 248 (98.8%) \| 249 (99.2%) \| 53 (96.4%) \| 52 (96.3%) \| 602 (98.5%) \| \| **RTC (items 1-10) total (10-50)** \|  \|  \|  \|  \|  \| \| Baseline \|  \|  \|  \|  \|  \| \| Mean (SD) \| 26.2 (6.2) \| 27.7 (6.4) \| 27.0 (7.3) \| 26.3 (6.8) \| 26.9 (6.5) \| \| Median (Q1, Q3) \| 26.0 (22.0, 30.0) \| 28.0 (23.0, 32.0) \| 27.0 (24.0, 31.0) \| 27.5 (20.0, 32.0) \| 27.0 (22.0, 32.0) \| \| Min, Max \| 10.0, 48.0 \| 10.0, 46.0 \| 12.0, 48.0 \| 13.0, 40.0 \| 10.0, 48.0 \| \| N (% Non-missing) \| 251 (100.0%) \| 251 (100.0%) \| 55 (100.0%) \| 54 (100.0%) \| 611 (100.0%) \| \| Midline \|  \|  \|  \|  \|  \| \| Mean (SD) \| 25.9 (6.2) \| 28.2 (6.2) \| 26.0 (6.3) \| 29.9 (5.8) \| 27.2 (6.3) \| \| Median (Q1, Q3) \| 26.0 (21.0, 30.0) \| 29.0 (24.0, 32.0) \| 25.0 (20.0, 30.0) \| 30.0 (26.0, 35.0) \| 28.0 (23.0, 32.0) \| \| Min, Max \| 10.0, 45.0 \| 12.0, 44.0 \| 12.0, 41.0 \| 15.0, 43.0 \| 10.0, 45.0 \| \| N (% Non-missing) \| 249 (99.2%) \| 250 (99.6%) \| 52 (94.5%) \| 53 (98.1%) \| 604 (98.9%) \| \| Endline \|  \|  \|  \|  \|  \| \| Mean (SD) \| 25.6 (5.5) \| 27.1 (6.2) \| 24.7 (5.4) \| 29.1 (6.5) \| 26.4 (6.0) \| \| Median (Q1, Q3) \| 25.0 (22.0, 29.0) \| 27.0 (23.0, 31.0) \| 24.0 (21.0, 29.0) \| 29.0 (26.0, 33.5) \| 26.0 (23.0, 31.0) \| \| Min, Max \| 13.0, 40.0 \| 10.0, 46.0 \| 14.0, 37.0 \| 14.0, 41.0 \| 10.0, 46.0 \| \| N (% Non-missing) \| 248 (98.8%) \| 249 (99.2%) \| 53 (96.4%) \| 52 (96.3%) \| 602 (98.5%) \| \| **Have you felt problems in your Heart mind?** \|  \|  \|  \|  \|  \| \| Baseline \|  \|  \|  \|  \|  \| \| Yes \| 251 (100.0%) \| 251 (100.0%) \| 55 (100.0%) \| 54 (100.0%) \| 611 (100.0%) \| \| Midline \|  \|  \|  \|  \|  \| \| No \| 55 (22.1%) \| 100 (40.0%) \| 17 (32.7%) \| 19 (35.8%) \| 191 (31.6%) \| \| Yes \| 194 (77.9%) \| 150 (60.0%) \| 35 (67.3%) \| 34 (64.2%) \| 413 (68.4%) \| \| Endline \|  \|  \|  \|  \|  \| \| No \| 70 (28.2%) \| 102 (41.0%) \| 22 (41.5%) \| 22 (42.3%) \| 216 (35.9%) \| \| Yes \| 178 (71.8%) \| 147 (59.0%) \| 31 (58.5%) \| 30 (57.7%) \| 386 (64.1%) \| \| **50% reduction in PHQ-9 from baseline to midline** \|  \|  \|  \|  \|  \| \| No \| 213 (85.5%) \| 185 (74.0%) \| 42 (80.8%) \| 44 (83.0%) \| 484 (80.1%) \| \| Yes \| 36 (14.5%) \| 65 (26.0%) \| 10 (19.2%) \| 9 (17.0%) \| 120 (19.9%) \| \| **50% reduction in PHQ-9 from baseline to endline** \|  \|  \|  \|  \|  \| \| No \| 208 (83.9%) \| 178 (71.5%) \| 41 (77.4%) \| 33 (63.5%) \| 460 (76.4%) \| \| Yes \| 40 (16.1%) \| 71 (28.5%) \| 12 (22.6%) \| 19 (36.5%) \| 142 (23.6%) \| |

**Table M: Mediation analyses**^e^ **for the primary outcome (GHQ at Endline with RTC at Midline)**

|  | **PM+ Effect on GHQ at Endline** | |  | **Midline RTC Effect** **on GHQ at Endline** | | | | | | | |
| --- | --- | --- | --- | --- | --- | --- | --- | --- | --- | --- | --- |
|  | **Mean Difference (95% CI)** | ***P*-value** |  | **RTC Effect** | |  | **Between-Ward RTC Effect** | |  | **Within-Ward RTC Effect** | |
|  |  |  |  | **Mean Difference (95% CI)** | ***P*-value** |  | **Mean Difference (95% CI)** | ***P*-value** |  | **Mean Difference (95% CI)** | ***P*-value** |
| **Endline-Only Analysis**^a^ | -1.3  (-2.5, -0.1) | 0.039 |  | - | - |  | - | - |  | - | - |
| **Endline-Only + Adjustment for RTC at Midline**^b^ | -1.1  (-2.4, 0.1) | 0.066 |  | -0.1  (-0.2, 0.0) | 0.146 |  | - | - |  | - | - |
| **Endline-Only + Adjustment for RTC at Midline using group-mean centering, or CWC(M)**^c^ | -0.9  (-2.2, 0.4) | 0.182 |  | - | - |  | -0.2  (-0.4, 0.0) | 0.104 |  | -0.0  (-0.1, 0.0) | 0.310 |

|  | **PM+ Effect on RTC at Midline** | |
| --- | --- | --- |
|  | **Mean Difference (95% CI)** | ***P*-value** |
| **Midline-Only Analysis**^d^ | 2.1 (0.9, 3.2) | 0.001 |

^a^ This model included these covariates: arm, ward gender (to account for the stratified design), the three covariates used in the constrained randomization procedure (i.e. access to mental health services, disaster risk and rural/urban status), each participant’s baseline measure of the outcome (GHQ), and each participant’s baseline measure of RTC. Note that it does not adjust for the mediator i.e. midline RTC.

^b^ One additional covariate, RTC at midline, together with all of the covariates mentioned above in ^a^ were included in the model. Note that we included this model in the results table since this is the commonly used model to generate an estimate of “ c’ ” to be used in the difference-in-coefficients approach to estimate the amount mediated by RTC. The difference-in-coefficients approach estimates the amount mediated using c – c’, where “c” is the estimated overall treatment effect (i.e. without adjustment by RTC at midline). In this case, “ c’ ” would be estimated using -1.1 with 95% CI of (-2.4, 0.1). However, Zhang et al (2009) showed that such a method may be biased and they instead recommended an alternative, namely to estimate “ c’ ” using a different model. Specifically, that model is the one referred to as “CWC(M)” with results in the next row and a more comprehensive description in the next footnote.

^c^ Two additional covariates - ward-mean of RTC at midline, difference between each participant’s RTC at midline and ward-mean of RTC at midline - together with all of the covariates mentioned above in ^a^ were included in the model. CWC(M) = centered within context with reintroduction of the subtracted means. Note that this is the recommended approach to mediation analysis for a cluster RCT with treatment assignment at “Level 2” (i.e. ward/cluster level) and both the mediator (RTC) and outcome at the participant-level (i.e. “Level 1”). It is described and recommended by Zhang et al. (2009), with details of the methods contained in Table 1 of their paper. Specifically, we used the recommended approach in the second set of equations numbered (1)-(4) and (8)-(10), where the estimated mediated effect is estimated by the difference-in-coefficients methods, namely c – c’ and where “c” is the estimated intervention effect from the model labeled “Endline-Only Analysis” (which corresponds to what is labeled (1)-(2) in Table 1 of Zhang et al) and where “ c’ ” is now estimated as the intervention effect from the model labeled “Endline-Only + Adjustment for RTC at Midline using group-mean centering, or CWC(M)”, namely -0.9 with estimated 95% CI of (-2.2, 0.4). In this model, we have also provided what are called the between-ward and within-ward effects of the mediator, namely RTC. We see that these are different from each other, which is further evidence that it is valuable to use the CWC(M) approach rather than the standard adjustment for RTC. In fact, as expected, the RTC effect on GHQ-12 estimated from the standard model labeled as “Endline-Only + Adjustment for RTC at Midline” is between these two values because the regular model constrains both the between- and within-ward effects of RTC to be equal and can lead to bias in estimating the effects mediated by RTC.

^d^ This model included these covariates: arm, ward gender (to account for the stratified design), the three covariates used in the constrained randomization procedure (i.e. access to mental health services, disaster risk and rural/urban status), each participant’s baseline measure of the outcome (RTC), and each participant’s baseline measure of GHQ.

^e^ Mediation effect (i.e. what portion of the PM+ effect on GHQ-12 at endline is estimated to be through RTC at midline) using the difference-in-coefficients method: from the commonly used procedure by comparing model ^b^ and model ^a^, the mediation effect is -0.1; from the group-mean centering or CWC(M) procedure by comparing model ^c^ and model ^a^, the mediation effect is -0.4. Note that, as described above, the preferred approach according to Zhang et al (2009) is to use the effect estimated from the CWC(M) approach, so that we would conclude that the mediation effect is -0.4. For the purposes of comparison, the mediation effect using the product-of-coefficients method is given as follows: from the commonly used procedure through model ^b^ and model ^d^, the mediation effect is -0.1; from the group-mean centering or CWC(M) procedure through model ^c^ and model ^d^, the mediation effect is -0.3. Note that we prefer the difference-in-coefficients approach as it is more intuitive to us. The results from the difference-in-coefficients and product-of-coefficients approach are not exactly equivalent in a cluster RCT with multilevel data although they would be expected to be in most cases in non-clustered data.

Reference: Zhang Z, Zyphur MJ, Preacher KJ (2009). Testing Multilevel Mediation Using Hierarchical Linear Models. Organizational Research Methods; 12(4): 695-719.
