# Supplementary material for: Effectiveness of Group Problem Management Plus, a brief psychological intervention for adults affected by humanitarian disasters in Nepal: A cluster randomized controlled trial
Source: PLoS Med. 2021 Jun 17;18(6):e1003621. doi: 10.1371/journal.pmed.1003621 (PMC8211182; doi:10.1371/journal.pmed.1003621)
Supplement: Attachment — Submitted filename: Review comments.edited.docx [file pmed.1003621.s004.docx]

**Review comments**

1. **Outcomes:** The study looked at psychological distress among participants in Nepal as the primary outcome of interest. The primary outcome was clearly stated and was properly measured by the General Health Questionnaire (GHQ) given that it has been validated in Nepal. I addition, this study measured several secondary outcomes, including depression, PTSD, "heart-mind problem", general functioning. From the instrument section, it is not clear if the perceived social support and somatic symptoms were considered as covariates or additional secondary outcomes of interest. This should well be elaborated. The three-time points for assessment (baseline, midline and end line) were properly designed and analyses followed the longitudinal nature of the study.

2. **Sample size:** The study provided a clear description of how the sample size was obtained, especially for the primary outcome at end-line timepoint. Alpha level was provided as 5%, the power to detect effect size, and attrition for both arms was provided.

3**. Sequence generation:** The study provided a clear description of the random allocation of the study participants to either comparison or intervention groups. Stratification and additional constrained were used to restrict randomization. Of note, the randomization was done at ward/cluster level and the analyses were done at the participant level.

4 & 5. **Allocation concealment and blinding**: The study described how they masked the researchers about the allocation of the participant to either enhanced usual care or group PM +. However, what was described as the strict separation between Assessors and Group PM+ facilitators, i.e. office separation and asking the research assistants to guess the allocation status of the study participants did not fit "strictness". I would suggest that the authors should admit the possibility of knowing the allocation status by the research assistants and put it as one of the limitations of the study.

6. **Outcomes and estimation:** The study reported an estimated effect size of 0.46 for the primary outcome. The means, standard deviations for each treatment arm and their corresponding mean differences and 95% Confidence intervals were appropriately reported. The study used risk ratio, risk differences in percentages points, corresponding 95% Confidence Intervals for both RR and RD.

7. **Harm**: the study used a DSMB and reported three serious adverse events (SAE) in the comparison group. No harms were attributed to the study intervention. It could be great if they can add more information to understand better what kind of help was extended to participants who had SAE.

8. **Registration:** the registry and registration number for this trial were provided.

9. **Protocol:** trial protocol was published.

**10. Funding:** The source of funding was reported as USAID and the US National Institutes of Health (NIH). The study described that the funders did not have any role in any aspect of the study.
