# Supplementary material for: Effectiveness of Group Problem Management Plus, a brief psychological intervention for adults affected by humanitarian disasters in Nepal: A cluster randomized controlled trial
Source: PLoS Med. 2021 Jun 17;18(6):e1003621. doi: 10.1371/journal.pmed.1003621 (PMC8211182; doi:10.1371/journal.pmed.1003621)
Supplement: Attachment — Submitted filename: 1_R1_Response to Reviewers_210113.docx [file pmed.1003621.s005.docx]

Requests from the editors:

Please remove the information on funding from the title page. In the event of publication, this information will appear in the article metadata via entries in the submission form.

AUTHORS’ RESPONSE: We have removed this.

Please revisit your data statement. We would view publication under a CC BY licence as open-access publication (similar to the licence used at PLOS). You appear to be indicating that display items will be made available in this way, however: are you able to share patient-level data in spreadsheet(s)? Please note that article authors cannot serve as points of contact for inquiries about data access according to PLOS' data policy.

AUTHORS’ RESPONSE: We are preparing the data for upload on Duke University’s Research Data Repository (<https://research.repository.duke.edu/>) as a .csv file along with an Excel file of the data dictionary. Both will be available online in the Duke Repository by **January 27**^th^. This will include all of the anonymized patient-level data to produce the main tables in the manuscript.

In your title, we suggest moving "Group Problem Management Plus" before the colon.

AUTHORS’ RESPONSE: We have changed the title to:

“Effectiveness of Group Problem Management Plus a brief psychological intervention for adults affected by humanitarian disasters in Nepal: a cluster randomized controlled trial”

Please adapt the abstract to the three-part PLOS Medicine style. The final sentence of the "Methods and findings" subsection should begin "Study limitations include ..." or similar and quote 2-3 of the study's main limitations.

AUTHORS’ RESPONSE: We have structured the abstract according to PLOS Medicine 3-part style. We have added the following as the last sentence of the methods and findings:

*“Study limitations include lack of power to evaluate gender-specific effects, lack of long-term outcomes (e.g., 12 months), and lack of cost-effectiveness information.”*

After the abstract, we will need to ask you to add a new and accessible "Author summary" in non-identical prose. You may find it helpful to consult one or two recent research papers in PLOS Medicine to get a sense of the preferred style.

AUTHORS’ RESPONSE: We have added the following author summary based on similar recent RCTs published in PLOS Medicine:

*AUTHOR SUMMARY*

***Why was this study done?***

- *Millions of people worldwide are affected by humanitarian emergencies such as war, environmental disasters, and pandemics. Most populations in these settings lack access to mental health services.*
- *People who are not mental health specialists have been trained to effectively deliver psychological treatments, including Problem Management Plus (PM+), which is a brief 5-session intervention. However, there has only been one prior study of a group-based format of PM+ delivered by non-specialists.*
- *As the use of non-specialists increases, there are new questions about how these psychological interventions work when delivered by someone who is not a mental health professional. Studying potential mechanisms of action could be instrumental to increasing effectiveness especially when scaling up.*
- *Therefore, this study evaluated the effectiveness of Group PM+ in a disaster-prone setting in Nepal with delivery of the intervention by briefly-trained non-specialists, and the researchers evaluated a potential mechanism of action involved in reducing psychological distress.*

***What did the researchers do and find?***

- *The researchers conducted a cluster randomized controlled trial in a disaster-prone region of Nepal. Community members with no prior mental health training received 10 days of foundational helping skills training and 10 days of Group PM+ facilitation training. Those achieving minimum competency in psychological intervention facilitation were selected to deliver Group PM+ in their communities.*
- *Proactive community detection led to identification of 643 adults with moderate psychological distress and functional impairment. Based on cluster randomization, 319 adults received the Group PM+ intervention, typically in groups of 6-8 people. A control group of 324 individuals were referred for mental health services at primary care facilities.*
- *Adults who received Group PM+ delivered by non-specialists in their communities showed greater reduction in psychological distress at 3 months after the intervention compared to adults who had been referred for other services. In addition, at 3 months after the intervention, approximately 1 out 3 adults in Group PM+ had a 50% reduction in depression symptoms, compared to 1 out 6 who were referred.*
- *Regarding how the intervention produced changes, the researchers found that a third of the differences between study arms was explained by participants’ use of psychosocial skills taught in Group PM+.*

***What do these findings mean?***

- *In humanitarian emergencies with a lack of mental health specialists, a 5-session group psychological treatment delivered by non-specialists can be used to modestly reduce psychological distress and depression symptoms.*
- *The benefits of Group PM+ are partly explained by the degree of psychosocial skill use being promoted through the intervention. Therefore, future use of the intervention should explore how to enhance practice of these skills in daily life.*
- *Further research is needed to evaluate how the impact of Group PM+ differs by gender because the study points towards potentially less benefit among men compared to women.*

In what form was informed consent obtained?

AUTHORS’ RESPONSE: We added the following sentence to the ethics section:

*“Because of low literacy levels, all participants were read the consent form by the research assistant and given an opportunity to discuss questions about the study with the research assistant.”*

Please substitute "sex" for "gender" where appropriate.

AUTHORS’ RESPONSE: We have retained gender because the group formation was based on self-identified gender, not sex at birth. With the group nature of the intervention, it was not considered appropriate to assign groups based on sex at birth because of the possibility that this was discordant with their social and community identity. It could introduce significant distress to have a person living in the community as a man or a woman to a group based on sex at birth that was not aligned with their current identity. Therefore, gender more accurately reflects the group formation and analyses. We have added the following text to participants section:

*“Participants were categorized by self-identified gender rather than sex at birth. This was because it was considered inappropriate to expect participants to join groups that were based on sex at birth when that was discordant with their social and community identity.”*

Please adapt reference call-outs throughout the manuscript to the following style: "... publicly available [4,17].", noting the absence of spaces within the square brackets.

AUTHORS’ RESPONSE: We have now reformatted the in-text citations according to PLOS Medicine guidelines.

In your reference list, please convert all italics to plan text. Where appropriate, 6 author names should be listed rather than 3, followed by "et al.".

AUTHORS’ RESPONSE: We have now reformatted the references according to PLOS Medicine guidelines.

Please convert the attached CONSORT checklist to a stand-alone file, referred to in your methods section ("See S1_CONSORT_Checklist" or similar). In the checklist, please refer to individual items by section (e.g., "Results") and paragraph number rather than by page or line numbers, as the latter generally change in the event of publication.

AUTHORS’ RESPONSE: We have created a stand-alone CONSORT checklist file and updated it with information to match the revised abstract and manuscript.

Comments from the reviewers:

*** Reviewer #1: 

**Review comments**

1. **Outcomes:** The study looked at psychological distress among participants in Nepal as the primary outcome of interest. The primary outcome was clearly stated and was properly measured by the General Health Questionnaire (GHQ) given that it has been validated in Nepal. I addition, this study measured several secondary outcomes, including depression, PTSD, "heart-mind problem", general functioning. From the instrument section, it is not clear if the perceived social support and somatic symptoms were considered as covariates or additional secondary outcomes of interest. This should well be elaborated. The three-time points for assessment (baseline, midline and end line) were properly designed and analyses followed the longitudinal nature of the study.

[RESPONSE] Thank you for pointing out that our assessment and analyses were well designed. Regarding the two measures of perceived social support and somatic symptoms, they are both secondary outcomes and are listed in this way on pg. 9 in the methods, and pg. 12 in the results section, as well as in Table 3 where we provide results of “secondary score outcomes”. We have pasted the relevant sections here:

Pg. 9 “Secondary outcomes include depression symptoms measured using the Primary Health Questionnaire (PHQ-9),26 also validated in Nepal;13 “heart-mind” problems;13 general functioning measured with the WHODAS;20 PTSD symptoms using an adapted 8-item Nepali version of the PTSD Checklist (PCL)27,28 based on longer versions previously used in Nepal;29-32 perceived social support using the Multi-dimensional Scale of Perceived Social Support (MSPSS);33,34 and the Somatic Symptom Scale 8 (SSS-8).35” on pg. 9

Pg. 12 in results: “Secondary outcomes. The Group PM+ arm was associated with lower depression symptoms (PHQ-9 mean difference=-1.0, 95% CI: -1.8, -0.1, p=0.028). Group PM+ was not associated with lower functional impairment (WHODAS mean difference=1.5, 95% CI: -3.4, 0.4, p=0.118), PTSD symptoms (PCL-C mean difference=-1.0, 95% CI: -2.2, 0.1, p=0.084), perceived social support (MSPSS mean difference=1.0, 95% CI: -0.3, 2.3, p=0.138), nor somatic symptoms (SSS mean difference=-1.0, 95% CI: -2.2, 0.2, p=0.105), see Table 3.”

Table 4 shows results of “secondary score outcomes” and both are listed below.

We have now also added these secondary outcomes to the abstract: “Secondary outcomes included depression symptoms, posttraumatic stress disorder (PTSD) symptoms, ‘heart-mind’ problems, social support, somatic symptoms and functional impairment.”

2. **Sample size:** The study provided a clear description of how the sample size was obtained, especially for the primary outcome at end-line timepoint. Alpha level was provided as 5%, the power to detect effect size, and attrition for both arms was provided.

[RESPONSE] Thank you, we are pleased that the sample size was well described.

3**. Sequence generation:** The study provided a clear description of the random allocation of the study participants to either comparison or intervention groups. Stratification and additional constrained were used to restrict randomization. Of note, the randomization was done at ward/cluster level and the analyses were done at the participant level.

[RESPONSE] We thank the reviewer for the feedback.

4 & 5. **Allocation concealment and blinding**: The study described how they masked the researchers about the allocation of the participant to either enhanced usual care or group PM +. However, what was described as the strict separation between Assessors and Group PM+ facilitators, i.e. office separation and asking the research assistants to guess the allocation status of the study participants did not fit "strictness". I would suggest that the authors should admit the possibility of knowing the allocation status by the research assistants and put it as one of the limitations of the study.

[RESPONSE] We thank the reviewer for raising this important point. We agree with identifying the risk of unblinding as a limitation. We have revised this text and removed the word ‘strict’:

“The study also included detailed documentation of services received by the control condition and reasons for dropout throughout the study, procedures to minimize and monitor unblinding, high retention, and rarity of missing data.”

We have also added the details on unblinding to the results section:

*"In order to assess the degree to which research assistants remained blind to the group allocation of the research participants, we asked them to report what type of service they thought each participant they interviewed received. After midline, in the PM+ group, research assistant guessed the wrong type of treatment or reported not to know in 97.4% of the interviews (5.6% were incorrect guesses of individual counseling, 0.7%, or medication, and 91.1% were ‘I don’t know’); for the EUC arm the research assistants guessed wrong or reported not to know in 92.0% of the interviews (incorrect guesses included 2.7% individual counseling, and 2.3% medication; 87.0% were ‘I don’t know’).”*

6. **Outcomes and estimation:** The study reported an estimated effect size of 0.46 for the primary outcome. The means, standard deviations for each treatment arm and their corresponding mean differences and 95% Confidence intervals were appropriately reported. The study used risk ratio, risk differences in percentages points, corresponding 95% Confidence Intervals for both RR and RD.

[RESPONSE] Thank you for highlighting that we were comprehensive in our reporting.

7. **Harm**: the study used a DSMB and reported three serious adverse events (SAE) in the comparison group. No harms were attributed to the study intervention. It could be great if they can add more information to understand better what kind of help was extended to participants who had SAE.

[RESPONSE] We have now included the followed-up care that was arranged for each of the three SAE cases. The following was added to the manuscript: “In all of three cases, a trained psychosocial counsellor visited the family multiple times, providing (grief) counselling and case management services.”

8. **Registration:** the registry and registration number for this trial were provided.

[RESPONSE] No response needed.

9. **Protocol:** trial protocol was published.

[RESPONSE] No response needed.

**10. Funding:** The source of funding was reported as USAID and the US National Institutes of Health (NIH). The study described that the funders did not have any role in any aspect of the study.

[RESPONSE] No response needed.

*** Reviewer #2: 

Statistical review

This paper reports a cluster randomised trial in Nepal investigating a brief psychological intervention for coping with emergency situations. The authors show a significant effect on the primary outcome, reproducing earlier trials. They also investigate the mechanism of action.

The statistical methods used are suitable and the trial is reported well. I have some minor comments on the reporting below.

1.      Abstract - this would be easier to follow if structured.

[RESPONSE] We have revised according to the 3-part PLOS Medicine structured abstract.

2.      Abstract - From just the abstract I found the timing of the endpoint hard to follow as it switches between 20 weeks past baseline and 3 months (presumably 20 weeks post baseline is 3 months post treatment). I would recommend keeping the phrasing consistent within the abstract.

[RESPONSE] We agree with the reviewer, and we have now harmonized this throughout the manuscript. We now refer to the timing of the assessments as follows: “Participants in both arms were assessed at baseline, midline (7-weeks post-baseline; after treatment in the experimental arm) and endline (20-weeks post-baseline, approximately 3 months post-treatment).”

3.      For at least the primary outcome I would recommend providing a p-value in addition to the confidence interval.

[RESPONSE] We have inserted the p-value provided in Tables 3 and 4 into the abstract for the reported outcomes.

“At 3-months, this effect is significant at the 5% level with an estimated GHQ-12 score of 1.4 units (95% CI: 0.3, 2.5; p = 0.014) lower in Group PM+ compared to control when adjusting for the baseline GHQ-12, SMD=-0.2 (95% CI: -0.4, -0.0).”

4.      I'd recommend clarifying psychosocial skill is measured in participants within both arms; if possible a bit more detail on what it is would be useful.

[RESPONSE] We agree with the reviewed that some more information about the assessment of ways of coping will be beneficial to the readers. We have added/adapted the following to the Instruments section of the paper:

“We developed a 10-item Reducing Tension Checklist (RTC), our measure of the intervention mechanism of action (see RTC Supplemental file). RTC measures the use of behavioural and psychosocial coping skills related to Group PM+ content, aiming to evaluate skill acquisition relevant for Group PM+. Each of the four active ingredients of Group PM+ (i.e., problem solving, seeking social support, managing problems, stress management) have multiple items in the RTC, with a total score indicating the level of combined skill acquisition. As the RTC was used among both Group PM+ and EUC participants, it is worded so that it is relevant for both groups.”

5.      Page 9: a couple of the secondary endpoints mentioned here are not mentioned in the abstract - I would recommend ensuring that all secondary endpoints reported in the paper are mentioned in the abstract.

[RESPONSE] We thank the reviewer for pointing out this omission. We have now corrected this in the Abstract, to read: “Secondary outcomes included depression symptoms, posttraumatic stress disorder (PTSD) symptoms, ‘heart-mind’ problems, social support, somatic symptoms and functional impairment.”

6.      Page 10: "Sub-group analyses" seems to refer to a analysis population rather than what typically would be understood by subgroup analyses. The statistical analysis plan refers to this as a completers population which I think is a better way of phrasing it.

[RESPONSE] Many thanks for highlighting a typographical error. You are absolutely right and we have edited the sentence:

“Sub-group analyses excluded intervention arm participants who attended fewer than 4 Group PM+ sessions (“non-completers”) whilst using data from all control arm participants”

to read:

“Completers analyses excluded intervention arm participants who attended fewer than 4 Group PM+ sessions (“non-completers”) whilst using data from all control arm participants.”

7.      I would recommend mentioning the gender subgroup analysis in the statistical section of the paper as this was pre-specified.

[RESPONSE] We agree and have inserted the following text in the methods section, in between the description of analysis of secondary outcomes and mediation analysis.

*“*Exploratory sub-group analyses assessed if there were different treatment effects according to sex and baseline depressive symptoms by separately including each of the two variables in the analysis model together with interactions between that variable and intervention arm and time-point.”

8.      Page 11 "Sensitivity analyses additionally adjusted for predictors of missing outcomes." - can these be added?

[RESPONSE] We have now added this as a note:

“Sensitivity analyses additionally pre-specified adjustment for predictors of missing outcomes (where we note that, in practice, none *were identified*)*.*”

Please see too the results section where we note that:

“There was minimal missing outcome data (98.8% completed endline). There was no indication of baseline covariates differing by study arm and midline or endline data availability so we did not perform other sensitivity analyses to account for missing outcome data (**Supplemental Tables 10 and 11**).”

9.      Page 11 "Number of PM+ skills used" - I found it a bit confusing that there are 5 skills, but the RTC checklist was 10 items - is the mediating variable actually some score from the checklist or a number between 0 and 5?

[RESPONSE] In response to this question, as well as the question #4 above, we have included the following to the Instruments section:

“We developed a 10-item Reducing Tension Checklist (RTC), our measure of the intervention mechanism of action (see RTC Supplemental file). RTC measures the use of behavioural and psychosocial coping skills related to Group PM+ content, aiming to evaluate skill acquisition relevant for Group PM+. Each of the four active ingredients of Group PM+ (i.e., problem solving, seeking social support, managing problems, stress management) have multiple items in the RTC, with a total score indicating the level of combined skill acquisition. As the RTC was used among both Group PM+ and EUC participants, it is worded so that it is relevant for both groups.”

10.     Page 12: "There was no evidence of an interaction between gender and the treatment effect; and there was no suggestion of a benefit of Group PM+ for men in gender-specific sub-analyses" - I would recommend phrasing the second part of the sentence differently; the effect of the intervention appears to be consistent across the gender subgroups, although not significant in the male subgroup (which would be quite an underpowered test given it's only 20% of the sample size).

[RESPONSE] We agree that the test would be very underpowered in the male subgroup and likewise that the test of interaction would have low power. We like the reviewer’s proposal to edit the second part of the sentence. Given that the estimated magnitude of treatment effects are smaller in men than women (with point estimates of -1.1 and -0.9 at midline and endline, respectively, versus -3.0 and -1.5 for women), we propose a slightly different phrasing as follows:

“There was no evidence of an interaction between gender and the treatment effect. In gender-specific sub-analyses, there was a smaller estimated benefit of Group PM+ for men and 95% confidence intervals spanned a between-arm difference of 0; this is partly explained due to only 20% of the sample being male."

11.     Page 13: I'm not too knowledgeable about mediation analysis so this may not be possible - can an uncertainty interval on the 31% proportion be provided?

[RESPONSE] We agree that it would be preferable to include some kind of uncertainty interval on the 31% proportion. We expect that some kind of delta method or bootstrapping would be applicable. Given that, to our knowledge, there is no validated approach available, we propose to not implement an unvalidated method and therefore have not edited the text. Please let us know if you discover that we are wrong and that there is, indeed, an acceptable approach as we would really like to be able to provide such an interval.

James Wason

*** Reviewer #3:

Thank you for the opportunity to review this manuscript describing a cluster randomized controlled trial evaluating the effectiveness of a brief psychological intervention for adults affected by humanitarian disasters in Nepal. This is a rigorous and well-conducted trial, and an excellent contribution to the global mental health literature, especially for advancing use of task sharing psychological interventions in humanitarian settings. This study is timely, and adds further impressive evidence in support use of the PM+ intervention. A few general comments to consider below:

- Were the facilitators compensated for delivering PM+? How were the facilitators recruited?

[RESPONSE] Thanks you for raising these important questions. The facilitators were indeed compensated and we followed a systematic process of recruitment of facilitators, using a set of predefined criteria. The following was added to the manuscript:

“The facilitators were recruited following a set of predefined criteria, including living in the communities where the project took place, not having received prior mental health training, have completed higher secondary school, as well as based on interviews demonstrating adequate communication skills and motivation to serve community people (van t Hof, 2020). The facilitators were compensated for the implementing the interventions (control and Group PM+).”

- Can more details about the credentials of the supervisors be provided? Were these members of the research team?

[RESPONSE] The supervisors are two staff members of the implementing organization, TPO Nepal. Both are trained psychologists and experienced counsellors. They were part of the project team, but only responsible for the implementation of the interventions (control and Group PM+). We have now included some information about the supervisors in the manuscript.

- The assessment of competency of the facilitators using ENACT is an important strength in this study, as well as the fidelity to PM+. One concern is the logistical challenges with competency rating, especially across so many sites. Can the authors comment on this and whether this poses challenges to scaling up the PM+ model following this trial?

[RESPONSE] We are pleased that the reviewer picked up on this point. We believe that the inclusion of the assessment of competency, as well as Group PM+ fidelity and mechanisms of action, need to be part of the future roll-out of Group PM+ (as well as similar interventions), as a way of monitoring the quality of implementation of the intervention. If such monitoring is not done at scale, we can simply not assume that the treatment effects that have been shown in this trial translate to real-world practice. While challenging, we are of the opinion that routine assessment of such quality of care indicators is feasible, as demonstrated in a recent 6-country project that exactly evaluated the integration of using the ENACT (and similar tools) as part of the implementation of evidence-based psychological treatment in LMICs. We have modified the text in the discussion as follows to address this:

*“Another study strength was measuring competency of facilitators. Because non-specialist interventions are delivered by different cadres across settings, ranging from college graduate nurses to persons with only a high-school education [44], it is important to establish minimum criteria on standardized competency measures across settings and facilitator types [45]. In this trial, we used ENACT, which is publicly available in a digital format through the WHO EQUIP project [46]. This By employing competency assessments, we excluded facilitators who had low skill levels, and supplemented skill gaps for those who were retained. Through the competency assessments, we were able to observe that some non-specialists achieved no or only a few minimal competencies prior to training (e.g., scores of 0-33% on the ENACT), but these individuals achieved competency on most skills by the end of training. By reporting competency levels achieved by facilitators in this trial, future programs implementing Group PM+ can compare the competency of their facilitators to determine if their skills are adequate for safe and effective intervention delivery [45]. If such monitoring is not done at scale, we cannot assume that the treatment effects that have been shown under research conditions will translate to real-world practice. Through the use of digital platforms to train raters and actors on competency evaluation and guidance on conducting competency-based training, there is growing feasibility and expertise for this to be possible [46].”*

- These findings are important, and add to the other recent trials of the PM+ program. Is there any plans to assess longer term outcomes and sustained benefits of this program beyond the 3-month follow up?

[RESPONSE] We agree with the reviewer that this is an important question. Our group does not currently have plans to assess longer term outcomes, but we agree that this is something that should be done. We have added the following to the discussion: “Future research should also investigate long-term (e.g., 12 months) benefits of Group PM+.“ We have also added this as a study limitation in the abstract.

- The benefits of PM+ are clear, though what are the expected challenges to implementation and sustained delivery of this program in routine settings? There is no mention of costs in this study. Were costs collected? If not, this should be highlighted as a limitation and important area to expand on this work for supporting adoption of this program within health systems globally.

[RESPONSE] We agree with the reviewer that assessment of costs, value for money or cost-effectiveness are important next steps. We do not believe it is a limitation of the current study, as it is was not part of the study objectives. We propose to include this as part of the research agenda for the future. This statement now reads: ““Future research should also investigate long-term (e.g., 12 months) benefits, as well we the cost-effectiveness, of Group PM+.“ We have also added lack of cost effectiveness information as a limitation in the abstract.

***
