# Supplementary material for: Effectiveness of Group Problem Management Plus, a brief psychological intervention for adults affected by humanitarian disasters in Nepal: A cluster randomized controlled trial
Source: PLoS Med. 2021 Jun 17;18(6):e1003621. doi: 10.1371/journal.pmed.1003621 (PMC8211182; doi:10.1371/journal.pmed.1003621)
Supplement: Attachment — Submitted filename: PMplus R2_Response to reviewers.docx [file pmed.1003621.s006.docx]

Requests from Editors:

Please finalize the information in your data statement (submission form).

Data are available at <https://doi.org/10.7924/r4gh9jq3g>

Please add a comma to your title after "Plus".

RESPONSE: The comma was added.

Please move the Acknowledgements section from the title page to the end of the main text; and the information on Ethics to your Methods section. The information on data availability and trial registration can be removed, as this is available elsewhere in the article or metadata.

RESPONSE: These changes have been made.

Please quote summary demographic information for participants in the "Methods and findings" subsection of your abstract.

RESPONSE: We added the following sentence: “The overall sample (N=611) had a median age of 45 years (range 18-91 years), 82% of participants were female, 50% had recently experienced a natural disaster, and 31% had a chronic physical illness.”

After quoting the primary endpoint findings in the abstract, we suggest amending the text to "Among the secondary endpoints, Group PM+ was associated at endline with a larger proportion of ..." or similar.

RESPONSE: We have added as follows: “Among the secondary outcomes, Group PM+ was associated at endline…”

Please adapt the "Conclusions" subsection of your abstract to the style: "In this study, we found that ... modestly reduced ...", or similar.

RESPONSE: We have revised: “In this study, we found that a 5-session group psychological treatment delivered by non-specialists modestly reduced psychological distress and depression symptoms in a setting prone to humanitarian emergencies. Benefits were partly explained by the degree of psychosocial skill use in daily life. To improve the treatment benefit, future implementation should focus on approaches to enhance skill use by PM+ participants.”

Please trim the "What did the authors do and find" subsection of your author summary to 3-4 points, and we suggest removing some of the practical details of the study (which are also described in the abstract).

RESPONSE: We remove two of the bullets.

Please update references 50 and 51.

RESPONSE: We have updated one of the references, which has now been published. We reached out to the editor of the other journal for the other manuscript to request the acceptance status. We can update this at the time of reviewing the proofs with the editors response.

Please rename Fig 1 "Participant flowchart ...".

RESPONSE: This has been corrected.

Comments from Academic editor:

This is a significantly improved manuscript, and the authors were responsive to all of the reviewer comments. This is a rigorous and well-conducted cluster RCT of the PM+ intervention in humanitarian settings in Nepal. I only have one remaining comment in relation to the interpretation of the mediation outcome, which I believe may benefit from further statistical review.

Referring to Table 4, it looks like the scores on the RTC mediator measure barely change within and between study arms, and it also looks as though the baseline scores on the RTC are considerably higher in the PM+ group compared to the control group. My first question is whether these baseline differences between groups were factored into the mediation analyses? Also, with such small changes in this measure, is it possible to rule out that this was due to chance as opposed to the effects of the intervention? I believe that this highlights an important potential mechanism for the PM+ intervention; though, the authors may overstate the importance of this finding. Moreover, the RTC measure does not appear to have been previously validated, and was developed only for this study. Therefore, I believe more caution is warranted for interpreting the mediation analysis findings throughout this manuscript.

RESPONSE:

Response: Many thanks for asking these clarifying questions so that we can ensure our manuscript best summarizes the methods and results of the mediation analyses.

Before addressing each question in turn, we would first like to acknowledge that the findings of the mediation should be taken in the context of the smaller than anticipated magnitude of effect of the intervention on the primary outcome of GHQ-12 at the primary timepoint (endline). Specifically, we powered the trial for a standardized effect size of 0.46 and, in practice, estimated a magnitude of 0.2

(95% CI: -0.4, -0.0), which corresponds to an unstandardized effect of -1.4 units (95% CI: -2.5, -0.3). As such, 31% of this relatively small PM+ effect on the primary outcome (endline GHQ-12) would, in an absolute sense, to be considered a small effect. As noted in the manuscript, the baseline levels of RTC were actually relatively high (i.e. meaning participants were already implementing key skills) and thus there was limited “room for improvement”. The range on the RTC is 0-40, so baseline averages indicate that the skills are, on average used between ‘sometimes’ and ‘most of the time’, leaning towards the latter.

Regarding the first question as to whether baseline differences between groups were factored into the mediation analyses: Yes, they were. Specifically, the two regression models that we fitted as part of the mean-centering mediation approach (each with endline GHQ-12 as the outcome) both included baseline RTC and baseline GHQ-12 as adjustment covariates. This was for two reasons: first, as for the primary outcome analysis, adjustment of the baseline measure of the outcome can gain power and therefore decrease the width of confidence intervals. Moreover, adjustment for RTC accounts for the chance imbalance of baseline RTC that we saw between arms, whereby mean RTC was slightly higher (i.e. slightly “better”) in PM+ than in control. This was pre-specified in our analysis plan prior to seeing the observed differences.

To address this point, we have done the following:

Edited the abstract: “Use of psychosocial skills at midline *was estimated* to explain 31% of the PM+ effect on endline GHQ-12 scores.”

Edited the methods section to include the following detail, if allowable due to word count: *“Specifically, the group-mean centering approach requires that two models are fitted. First, endline GHQ-12 was regressed on the following covariates: arm, ward gender (to account for the stratified design), the three covariates used in the constrained randomization procedure (i.e. access to mental health services, disaster risk and rural/urban status), each participant’s baseline measures of both RTC and of GHQ; a random intercept for ward was included, with different ICC for each arm. A second model was fitted with two additional variables: the ward-level mean RTC and each participant’s RTC deviation from the ward mean. From these two models, the mediated effect was estimated as per Zhang et al. [45].*

Edited the results paragraph for the mediation analyses to read: *“Mediation analyses: After adjustment for chance imbalance in baseline levels of the mediator (i.e. skill use measured with the RTC), the hypothesized mediator was greater in the Group PM+ arm at midline (RTC mean difference=2.1, 95% CI: 1.0, 3.2, p<0.001) and endline (RTC mean difference=1.5, 95% CI: 0.3, 2.7, p=0.016), see* ***Table 4****. The corresponding estimated standardized effects were slightly smaller than for the primary outcome (****Tables 3 and 4****). The group-mean centering mediation analysis of RTC at midline as a mediator of GHQ-12 at endline, shows an estimated mediation effect of -0.4 units relative to the estimated intervention effect of -1.3 units (see* ***Figure 3****) so that the estimated relative portion of the Group PM+ effect on endline GHQ-12 that is mediated by midline RTC is 31% (****Supplemental Table 13****). ”*

Regarding the question as to whether it is possible to rule out that the estimated mediating effect of RTC was due to chance as opposed to the effects of the intervention: We agree that this is not possible and, indeed, it would not be possible even were there to be stronger effects in the study. As the reviewer agrees, RTC is a potential mechanism for the PM+ intervention. We understand the reviewer’s concerns about how best to state the importance of this finding.

As a consequence, we have added the following text to the limitations paragraph: *“Although we used the mean-centering mediation method that avoids the bias that can arise in the traditional non-centered approach, there are several limitations to our approach. First, as with all mediation analyses, bias may arise if confounders of the mediator (midline RTC) and outcome (endline GHQ-12) relationship are missing from the model. Second, with smaller than anticipated overall effects of PM+ on endline GHQ-12, our estimation that 31% of that effect was mediated by midline RTC must be interpreted relative to the overall magnitude of effect. Relatedly, future studies should also consider more objective measures of mechanisms of action that are less subject to self-report bias.”*

Regarding the point that the RTC measure does not appear to have been previously validated, and was developed only for this study so that more caution is warranted for interpreting the mediation analysis findings throughout this manuscript, we have added the following text: “Regarding mediation, the tool used to evaluate mechanisms of action, the RTC, was developed and piloted during the preceding feasibility study. However, the RTC has not been validated in other populations.”

One minor point, in the Instruments section on page 12, the authors refer to the PHQ-9 as the "Primary Health Questionnaire" for measuring depression. This should be changed to "Patient Health Questionnaire".
RESPONSE: This has been corrected.

Comments from Reviewers:

*** Reviewer #2:

Thank you to the authors for addressing my previous comments well. I agree with their responses and have no further issues to raise.
